# Transgenic *Anopheles* mosquitoes expressing human PAI-1 impair malaria transmission

**DOI:** 10.1038/s41467-022-30606-y

**Published:** 2022-05-26

**Authors:** Tales V. Pascini, Yeong Je Jeong, Wei Huang, Zarna R. Pala, Juliana M. Sá, Michael B. Wells, Christopher Kizito, Brendan Sweeney, Thiago L. Alves e Silva, Deborah J. Andrew, Marcelo Jacobs-­Lorena, Joel Vega-Rodríguez

**Affiliations:** 1 Laboratory of Malaria and Vector Research, National Institute of Allergy and Infectious Diseases, National Institutes of Health, 12735 Twinbrook Parkway, Rm 2E20A, Rockville, MD 20852 USA; 2 Department of Molecular Microbiology and Immunology, Malaria Research Institute, Johns Hopkins Bloomberg School of Public Health, Baltimore, MD 21205 USA; 3 Department of Cell Biology, Johns Hopkins University School of Medicine, 725 N. Wolfe Street, G10 Hunterian, Baltimore, MD 21205 USA; 4 Present Address: Department of Biomedical Sciences, Idaho College of Osteopathic Medicine, Meridian, ID 83642 USA

**Keywords:** Parasite biology, Entomology, Extracellular matrix

## Abstract

In mammals, the serine protease plasmin degrades extracellular proteins during blood clot removal, tissue remodeling, and cell migration. The zymogen plasminogen is activated into plasmin by two serine proteases: tissue-type plasminogen activator (tPA) and urokinase-type plasminogen activator (uPA), a process regulated by plasminogen activator inhibitor 1 (PAI-1), a serine protease inhibitor that specifically inhibits tPA and uPA. *Plasmodium* gametes and sporozoites use tPA and uPA to activate plasminogen and parasite-bound plasmin degrades extracellular matrices, facilitating parasite motility in the mosquito and the mammalian host. Furthermore, inhibition of plasminogen activation by PAI-1 strongly blocks infection in both hosts. To block parasite utilization of plasmin, we engineered *Anopheles stephensi* transgenic mosquitoes constitutively secreting human PAI-1 (huPAI-1) in the midgut lumen, in the saliva, or both. Mosquitoes expressing huPAI-1 strongly reduced rodent and human *Plasmodium* parasite transmission to mosquitoes, showing that co-opting plasmin for mosquito infection is a conserved mechanism among *Plasmodium* species. huPAI-1 expression in saliva induced salivary gland deformation which affects sporozoite invasion and *P. berghei* transmission to mice, resulting in significant levels of protection from malaria. Targeting the interaction of malaria parasites with the fibrinolytic system using genetically engineered mosquitoes could be developed as an intervention to control malaria transmission.

## Abstract (web summary)

*Plasmodium* gametes and sporozoites activate surface-bound plasminogen to plasmin that degrades extracellular matrix barriers, therewith facilitating parasite motility in mosquitoes and mammalian hosts. To control malaria transmission, Pascini et al. generate *Anopheles stephensi* transgenic mosquitoes constitutively secreting human plasminogen activator inhibitor 1 in midgut and/or saliva which leads to inhibition of plasminogen activation and a reduction in oocyst intensity, infection prevalence, and transmission.

## Introduction

Malaria, a mosquito-borne infectious disease, is a global public health threat that, in 2019, infected 228 million people and resulted in ~409,000 deaths^1^. Malaria is caused by *Plasmodium* parasites and is transmitted by the bite of an infected *Anopheles* mosquito. The complex life cycle of the parasite in the mosquito and the vertebrate host makes malaria an extremely challenging disease to control.

In mosquitoes, after the ingestion of a *Plasmodium*-infected blood meal, the parasite’s male microgamete must migrate through the compacted blood bolus in the mosquito midgut lumen to fertilize a female macrogamete and produce a zygote^2^. Zygotes differentiate into motile ookinetes that migrate through the blood bolus, traverse the peritrophic matrix and the midgut epithelium, developing into oocysts on the basal side of the midgut. When mature, each oocyst releases thousands of sporozoites into the hemolymph from where they invade the mosquito salivary glands^3^. During blood feeding, an infected mosquito releases saliva and sporozoites into the human dermis from where they must migrate to invade a blood vessel and enter the circulation^4^. Sporozoites exit the circulation in the liver and migrate into the hepatic tissue to infect hepatocytes, multiply and differentiate into merozoites that are released back into circulation to start the erythrocytic phase^5^. Throughout its life cycle, the parasite must overcome several physical barriers including fibrin networks, extracellular matrices, and epithelial and endothelial cell barriers to establish an infection in both the mosquito and the human. Previously, we showed that parasite migration through some of these barriers is facilitated by components of the mammalian fibrinolytic system^6,7^.

In mammals, the fibrinolytic system is essential for several physiological processes, including homeostasis of blood coagulation, degradation of blood clots, cell migration, and embryogenesis^8^. Fibrinolysis is facilitated by the catalytic action of plasmin, a crucial serine protease that degrades fibrin and other extracellular matrix proteins. Plasmin is activated from its zymogen plasminogen via proteolytic cleavage by the serine proteases tissue-type plasminogen activator (tPA) and urokinase-type plasminogen activator (uPA). Plasminogen activation is mainly regulated by plasminogen activator inhibitor 1 (PAI-1), a serine protease inhibitor (serpin) that binds covalently to the active site of tPA and uPA and inhibits their protease activity^9,10^. Plasminogen activator inhibitor 2 (PAI-2) can also regulate fibrinolysis, although it has 10 times less inhibitory activity against uPA, minimal activity against single-chain tPA (proenzyme), and is not fully secreted^11^.

Previously, we showed that *Plasmodium* mosquito midgut stages and sporozoites bind plasminogen, tPA, and uPA to their surface where plasminogen is activated into plasmin^6^. Surface plasmin is used by the gametes to degrade the fibrin network that polymerizes in the midgut blood bolus upon blood feeding, facilitating parasite migration in the mosquito midgut^6,7^. Likewise, sporozoite-bound plasmin facilitates migration through the extracellular matrices of the dermis and the liver, allowing for the establishment of a liver infection^6,7^. Since activation of parasite-bound plasminogen is mediated by co-recruited mammalian tPA and uPA, plasminogen activation and parasite infection of both mosquito vector and mammalian host are inhibited in the presence of PAI-1^6^. These findings highlighted the importance of the fibrinolytic proteins for parasite infectivity and suggest a potential strategy to block malaria transmission.

In recent years, the reduction of mosquito vectorial capacity through genetic modification has shown great potential for malaria intervention. Mosquito transgenesis to control malaria focuses on two main approaches: population suppression to eradicate or reduce mosquito numbers; and population modification, in which the wild type population is rendered refractory to the infection by either expressing anti-plasmodial molecules or modifying mosquito genes essential for parasite transmission^12–14^. In the last decade, much progress has been made in the development of transgenic mosquitoes refractory to malaria parasites. This includes modifying mosquito genes essential for parasite development^15–17^, enhancing mosquito immune factors^18–20^, expression of antiparasitic toxins or molecules^20–24^, and expression of single-chain antibodies targeting parasite proteins^25,26^.

In this study, we report the engineering of transgenic *Anopheles* mosquitoes that constitutively express human PAI-1 (huPAI-1) in the midgut and/or salivary glands in order to target plasminogen activation at the surface of the malaria parasite. In this approach, the parasite is still able to bind plasminogen, but its activation is blocked by PAI-1 secreted in the midgut lumen where it targets the parasite sexual stages, or into the saliva where it targets the sporozoites as well as the midgut stages. We observe a potent inhibition of *P. berghei*, *P. falciparum*, and *P. vivax* infections in the transgenic mosquitoes, and a strong reduction of *P. berghei* transmission to the mammalian host. Expression of huPAI-1 does not cause a fitness cost on mosquito survival, fertility, and fecundity. Here, we report the use of transgenic *Anopheles* mosquitoes expressing a human protein to thwart parasite development in the vector and mammalian host.

## Results

### Recombinant huPAI-1 inhibits *P. falciparum* infection of *An. stephensi*

We previously reported that supplementation of a *P. falciparum* infectious blood meal with huPAI-1 inhibits plasminogen activation and oocyst formation in *An. gambiae* mosquitoes^6^. To determine if huPAI-1 inhibition of plasminogen activation also inhibits *P. falciparum* infection of *An. stephensi* mosquitoes, we performed standard membrane feeding assays (SMFAs) with *P. falciparum* gametocytes supplemented with plasma and increasing concentrations of huPAI-1 (0–25 μg/mL). Microscopic examination of mosquito midguts dissected 7–8 days post-infection show that huPAI-1 inhibits *P. falciparum* oocyst formation and reduces the prevalence of infection (Supplementary Fig. 1a and Supplementary Data 1, Dataset 1). huPAI-1 inhibition of infection was restored by addition of plasmin (Supplementary Fig. 1b and Supplementary Data 1, Dataset 2). These results indicate that *P. falciparum* hijacks plasmin for infection of both *An. gambiae* and *An. stephensi*.

### Generation of *An. stephensi* mosquitoes expressing huPAI-1

**Fig. 1:**
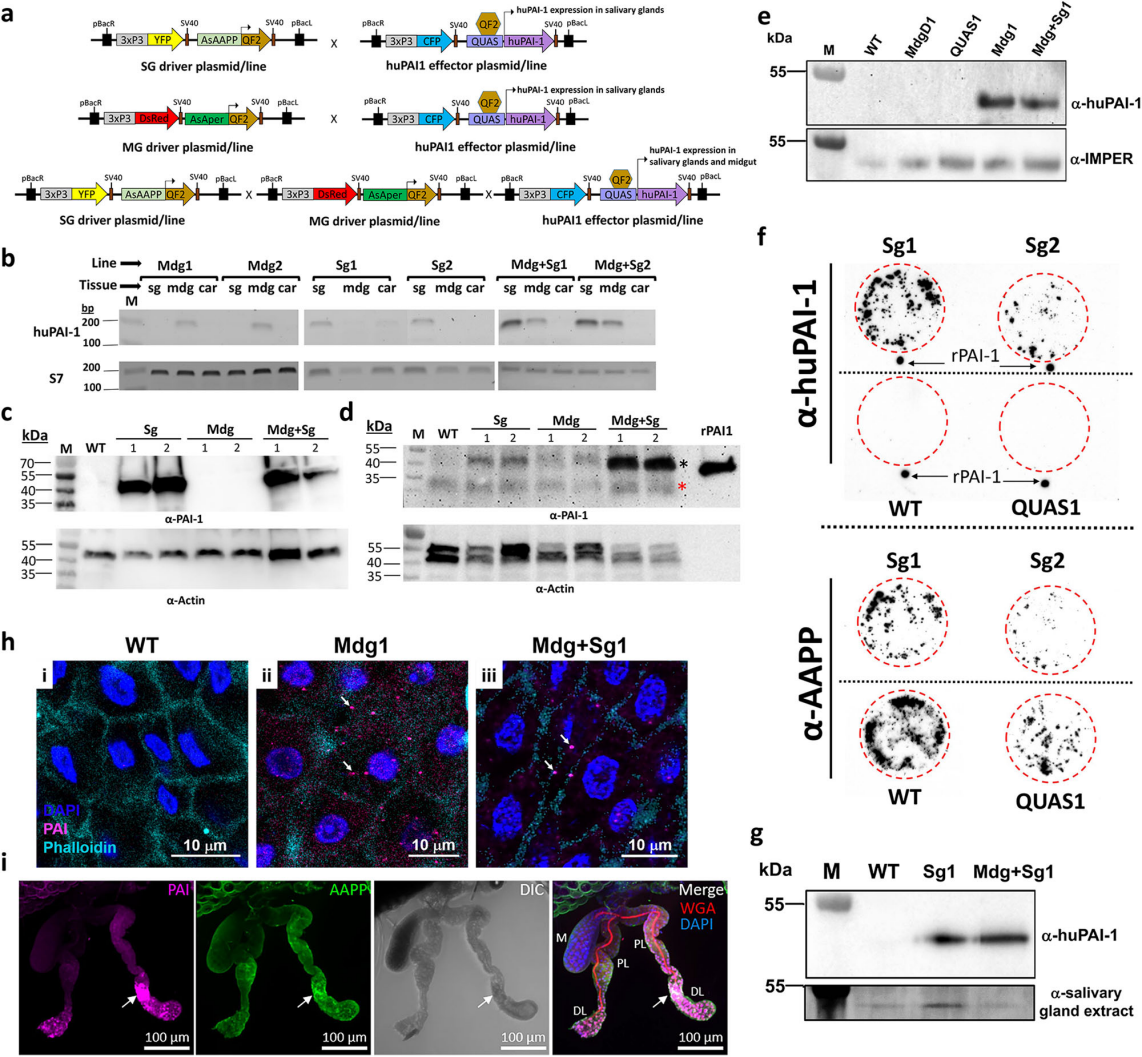
Tissue-specific expression of huPAI-1 in transgenic *An. stephensi* mosquitoes. **a** Schematic of the plasmids used to develop the driver and effector parental lines. In the driver lines, the QF2 transcription factor is expressed by the AsAAPP promoter or the AsAper promoter in the salivary glands or the midgut, respectively. Crossing the driver and the effector lines (Supplementary Fig. 2c) induces salivary gland- (SG) and/or midgut- (MG) specific expression of human PAI-1 (huPAI-1). Transgene integration in the genome was mediated by the inverted terminal repeats of piggyBac (pBacR and pBacL). SV40: transcription terminator sequence. **b** Tissue-specific expression of huPAI-1 mRNA detected by RT-PCR in the midgut (mdg) and salivary glands (sg) of transgenic female mosquitoes. Carcasses (car) were used as negative controls. The ribosomal protein S7 was used as positive control. Mdg1 and Mdg2: huPAI-1 midgut transgenics, Sg1 and Sg2: huPAI-1 salivary gland transgenics, Mdg + Sg1 and Mdg + Sg2: huPAI-1 midgut and salivary gland transgenics. **c**, **d** Immunoblotting showing huPAI-1 protein expression (47 kDa) in salivary gland (**c**) and midgut (**d**) lysates from transgenic lines. Recombinant huPAI-1 (rPAI-1) was used as a positive control. WT wild type, M molecular size marker. The black asterisk in (**d**) points to the huPAI-1 specific bands and the red asterisk points to a non-specific band. **e** Midgut lumen huPAI-1 secretion was assessed by the low-melting agarose assay. Liquid low-melting agarose was fed to mosquitoes and after solidification, secreted proteins trapped in the midgut agarose bolus were analyzed by Western blotting with an anti-huPAI-1 antibody. An α-IMPer antibody was used as a positive control for a midgut secreted protein. M marker, MdgD1 midgut QF2 parental line, QUAS1 QUAS-huPAI-1 effector line. **f** “Spit-blot assay” showing secretion of huPAI-1 in the saliva. Mosquitoes were allowed to probe on a pre-heated nitrocellulose membrane. Membranes were analyzed by immunoblotting with an α-huPAI-1 antibody or an anti-AAPP antibody as a positive control. WT and QUAS1 parental mosquitoes were used as negative controls. Red-dashed circles show the probing area. Recombinant huPAI-1 was spotted outside the probing area as a positive control. **g** huPAI-1 is detected in the midgut lumen of Sg1. Ingestion of saliva containing huPAI-1 was confirmed by low melting agarose assay, followed by immunoblotting with an α-huPAI-1 antibody. An α-salivary gland extract antibody was used as a control for ingested saliva proteins. M marker. **h** Detection of huPAI-1 in the midgut of the WT (i), Mdg1 (ii), and Mdg + Sg1 (iii) lines. arrows: huPAI-1 staining in cytoplasm. **i** Localization of huPAI-1 expression in salivary glands of one-day-old Sg1 females stained with DAPI (DNA, blue), WGA (red), anti-AAPP (green) and anti-PAI-1 (purple). AAPP and PAI-1 signals localized throughout the salivary glands, with the highest levels observed in the proximal portion of the distal lateral lobes (arrow). DL distal lateral, M medial, PL proximal lateral. Data presented in panels (**b**–**i**) are representative of at least two independent experiments. Source data are provided as a Source Data file.

To constitutively express huPAI-1 in the mosquito midgut and salivary glands, we used the QF2-QUAS binary expression system previously adapted for expression in *An. gambiae*^27,28^. The “QF2-driver” plasmids consisted of either the *An. stephensi* anopheline anti-platelet protein (AAPP) salivary gland-specific constitutive promoter^29^, or the midgut-specific constitutive promoter for the adult peritrophin 1 (Aper1) gene^30,31^, inserted upstream of the QF2 transcription factor coding sequence^27^ (Fig. 1a). The QUAS “effector” plasmid contains the huPAI-1 coding sequence, including the endogenous huPAI-1 secretion signal, downstream of the QUAS enhancer (Fig. 1a)^27^. The huPAI-1 sequence contains the stabilizing mutations N150H, K154T, Q319L, and M354I that increase the protein half-life from 2 to 147 h^32^. The three plasmids have the eye-specific 3xP3 promoter driving the expression of the dsRed (red eyes, midgut QF2-driver), the CFP (blue eyes, QUAS effector), or the YFP (yellow eyes, salivary gland QF2-driver) selection markers (Fig. 1a).

The two driver plasmids and the effector plasmid were individually injected into *An. stephensi* embryos together with a helper plasmid for expression of a piggyBac transposase^33^. Transgene integration into the mosquito genome was mediated by the piggyBac transposable elements contained within the effector and driver plasmids. One parental line with stable expression of the fluorescence marker was generated for the midgut QF2-driver named MdgD1, and two parental lines were generated for the salivary gland drivers named SgD1 and SgD2 and for the QUAS-effectors named QUAS1 and QUAS2. Genome integration sites were determined for each parental line by splinkerette PCR^34^ and sequencing (Supplementary Fig. 2a, b). Except for SgD2, all the parental lines had insertions in intergenic regions. SgD2 had two transgene insertions in intergenic regions, and one integration in the open reading frame of the gamma-glutamyltranspeptidase gene (ASTE010947) (Supplementary Fig. 2b).

To induce huPAI-1 midgut- and/or salivary gland-specific expression, the QF2-driver lines were crossed with the huPAI-1 effector lines and selected based on eye marker color (Supplementary Fig. 2c). The resulting lines were named Mdg1 and Mdg2 for huPAI-1 expression in the midgut, and Sg1 and Sg2 for huPAI-1 expression in the salivary glands. To induce huPAI-1 expression in both midgut and salivary gland, we crossed the huPAI-1 midgut lines with the huPAI-1 salivary gland lines. The resulting lines were named Mdg + Sg1 and Mdg + Sg2 (Supplementary Fig. 2c).

### huPAI-1 is expressed and secreted in a tissue-specific manner

huPAI-1 gene expression in the transgenic mosquitoes was assayed by reverse transcription-polymerase chain reaction (RT-PCR). Expression was only detected in the midguts of the Mdg1 and Mdg2 lines, in the salivary glands of the Sg1 and Sg2 lines, and in the midgut and salivary glands of the Mdg + Sg1 and Mdg + Sg2 lines (Fig. 1b). No RT-PCR signal was detected in any of the analyzed tissues of the WT line. Western blotting analysis using an anti-huPAI-1 antibody confirmed tissue-specific expression of the huPAI-1 protein (~47 kDa) (Fig. 1c, d). Based on the amount of total protein used for the Western blots (10 µg for salivary glands vs 40–50 µg for midguts), these data show that huPAI-1 expression by the AAPP promoter was stronger in the salivary glands than expression by the Aper promoter in the midgut. The huPAI-1 protein was detected in the midgut of the Sg lines (Fig. 1d), which can be attributed to the saliva ingested by the mosquito during sugar feeding^35^. This is supported by the absence of huPAI-1 mRNA expression in the midgut of the Sg mosquitoes (Fig. 1b).

The huPAI-1 coding sequence used for transgenic expression contained the endogenous secretion signal peptide. We predicted that PAI-1 would be secreted into the midgut lumen of the Mdg transgenic lines, and in the saliva of the Sg transgenics. Detection of huPAI-1 secretion into the midgut lumen was confirmed by a low-melting agarose assay^36^. In this assay, low melting agarose in its liquid state is fed to mosquitoes simulating the ingestion of a blood meal. Once the agarose solidifies in the midgut lumen it continuously absorbs the proteins secreted by the midgut epithelium, in this case, huPAI-1. The agarose bolus was dissected from the mosquito midgut and processed for Western blotting analysis. Secreted huPAI-1 was detected in the midgut lumen of the Mdg transgenic lines but not in the midgut of WT nor in the midguts of the parental MdgD1 and QUAS1 lines (Fig. 1e). An antibody against the midgut secreted protein heme peroxidase (IMPer), a protein previously shown to be secreted into the midgut lumen^37^, was used as a positive control (Fig. 1e). Western blot analysis of the agarose bolus with an antibody against the eNOS, a non-secreted midgut protein, confirms that the huPAI-1 detected in the agarose bolus of the Mg transgenics is secreted and not a contaminant from epithelial cells that remained attached to the agarose bolus after dissection (Supplementary Fig. 3).

**Fig. 2:**
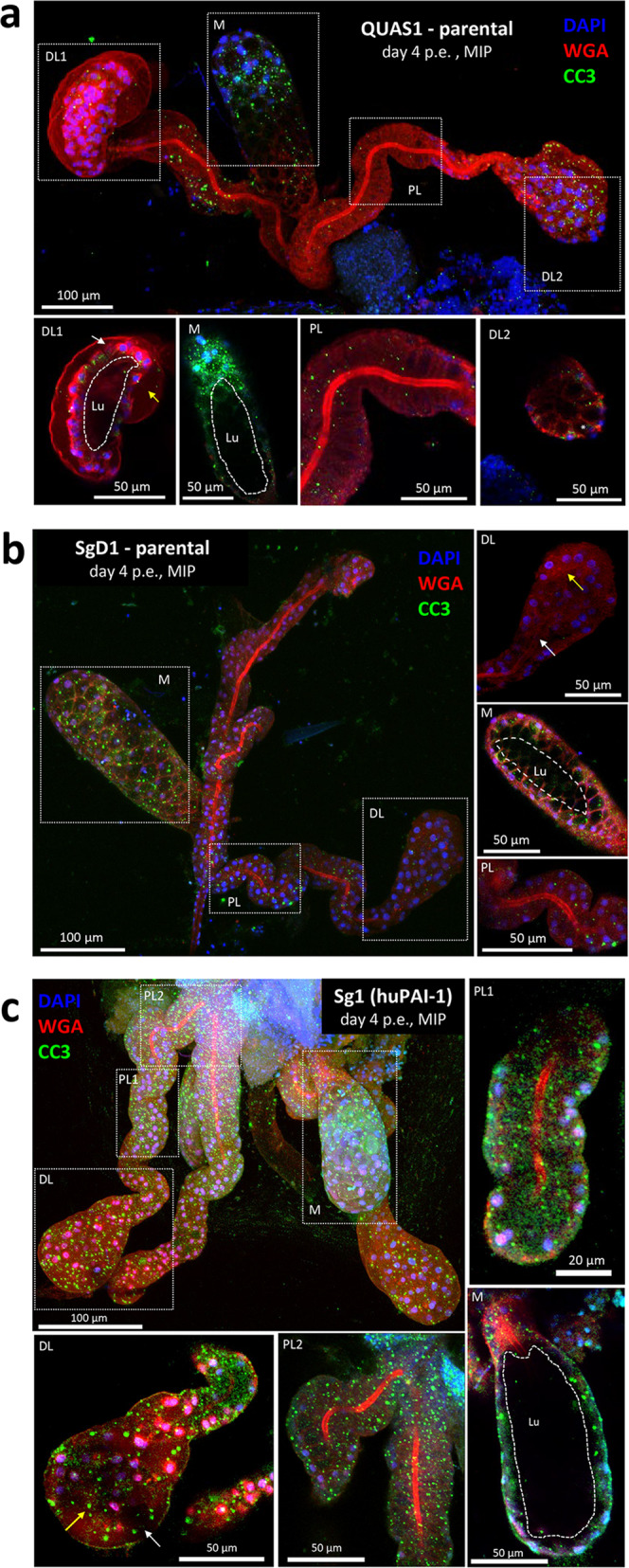
Expression of huPAI-1 in salivary glands is associated with architectural changes and increased cleaved caspase 3 signal. Salivary glands were dissected four days post-emergence (p.e.) and stained with anti-cleaved caspase 3 antiserum (CC3, a cell death marker; green), DAPI (DNA, blue), and WGA (chitin/O-GlcNAcylation; red). Shown are representative 3D maximum intensity projection (MIP) or central single slice confocal images of salivary glands from parental QUAS1 effector (**a**), parental SgD1 (**b**), and Sg1 transgenic (**c**) mosquitoes. When compared to QUAS1, salivary glands of mosquitoes expressing huPAI-1, or QF2 to a lesser extent, show morphological defects including loss of cells’ cup shape, loss of organization [a single cell layer surrounding a clearly defined lumen (dotted white line, Lu)], and increased staining for the cell death marker CC3. Medial lobes from all three strains are often positive for CC3 staining. A missing cell was identified in a-DL1 (white arrow) that explains the lumenal leakage behind the secretory cells of that DL lobe (yellow arrow). The asterisk in a-DL2 inset marks a secretory cavity that is similarly visible in the MIP image. The white arrow in b-DL marks the salivary duct terminus. Yellow arrows in b-DL and c-DL indicate sites of secretory cavities and cell misorganization (compare to a-DL1 and a-DL2). White arrow in c-DL shows a site of SG cell detachment from the basement membrane. Data presented in panels a-c are representative of at least two independent experiments. Additional examples of salivary glands from all three genotypes can be found in Supplementary Fig. 3.

Detection of huPAI-1 secretion into the saliva was analyzed by a “spit blot assay”. In this assay, mosquitoes probe on a cellulose membrane pre-warmed at 37 °C. huPAI-1 protein deposited in the membrane during probing was identified with an anti-huPAI-1 antibody. The same membrane was subsequentially incubated with an antibody against the saliva protein AAPP as a positive control for salivation. Both Sg1 and Sg2 mosquitoes deposited saliva containing huPAI-1 proteins while WT and QUAS1 control mosquitoes did not (Fig. 1f). Next, we examined if huPAI-1 expressed in the salivary glands is ingested together with the saliva in the Sg transgenic mosquitoes by using a low melting agarose assay. huPAI-1 was detected in the midgut of Sg1 and Mdg + Sg1 transgenics and not in the midgut of WT mosquitoes (Fig. 1g). These experiments suggest that the huPAI-1 protein detected in the midgut of Sg transgenics (Fig. 1d, g) in the absence of mRNA expression (Fig. 1b) originates from ingested saliva^35^. We also analyzed the expression of huPAI-1 in the midgut and salivary glands by immunofluorescence assay (IFA). Expression of huPAI-1 was detected in midguts of Mdg1 and Mdg + Sg1 transgenic mosquitoes in discrete puncta (arrows) that could represent secretory vesicles (Fig. 1h). Expression of huPAI-1 was also confirmed by IFA in the salivary glands of the Sg1 transgenic mosquitoes (Fig. 1i). At one day post-emergence, AAPP, and huPAI-1 signals were observed throughout the salivary glands, but most intense in the proximal portion of the distal lobes (DL) lobes (Fig. 1i, arrow). Salivary gland expression of huPAI-1 was assessed at one day post-emergence when the structural differences between WT and transgenic are minimal, as opposed to later time points (i.e., 4 and 14 days post-emergence) when a continuous deterioration of the transgenic salivary gland structure is observed (Fig. 2 and Supplementary Fig. 4e). Altogether, these results confirm tissue-specific expression and secretion of huPAI-1 in transgenic mosquitoes.

### huPAI-1 expression is associated with architectural changes in salivary glands, but not in midguts

The structural integrity and viability of the midgut and salivary gland epithelium in mosquitoes expressing huPAI-1 was evaluated by IFA. Midguts from WT, Mdg1, and Mdg + Sg1 mosquitoes were stained for actin (Phalloidin), DNA (DAPI), and with an anti-huPAI-1 antibody. No evidence of tissue deformation was observed in the midguts of transgenic mosquitoes when compared to WT. Cell morphology indicated by phalloidin stain shows a similar cell shape of Mdg1 and Mdg + Sg1 lines when compared to the WT (Fig. 1h).

To determine the structural integrity of salivary glands expressing huPAI-1, salivary glands were dissected from Sg1 transgenics, and SgD1 and QUAS1 parental adults four days post-emergence, when adult salivary glands are morphologically mature^38^. Salivary glands were stained with DAPI (DNA; blue), WGA (O-GlcNAcylation/Chitin; red), and antisera against cleaved caspase 3 (CC3, cell death marker; green). The QUAS1 parental salivary glands were WT in appearance (Fig. 2a)^38,39^. All cells were organized in a single monolayer around a salivary duct and/or central salivary gland lumen (Lu) in all three lobes (Fig. 2). Cell secretory cavities were readily visible (Fig. 2a-DL2 asterisk). CC3 staining was minimal in the distal lateral (DL) and proximal (PL) lobes (Fig. 2a-DL1, DL2, and PL) but concentrated in perinuclear foci in the median (M) lobe (Fig. 2a-M). In the salivary glands of the parental SgD1 line (Fig. 2b), DL lobes nearly always lacked a lumen, with cells filling the central interior of the lobe (Fig. 2b-DL, yellow arrow). In contrast, the M and PL lobes (Fig. 2b-M and PL) appeared very similar to the QUAS1 parental salivary glands. The expression of the QF2 transcription factor is known to be slightly toxic^40^ and its expression in DL lobes may be related to lumen loss. Finally, salivary glands of the Sg1 adults expressing huPAI-1 (Fig. 2c) showed the strongest phenotype, with DL lobe lumen loss (Fig. 2c-DL), DL lobe cell secretory cavity loss and mis-organization (2c-DL, yellow arrow), and cell bodies that had pulled away from the basement membrane (Fig. 2c-DL, white arrows). The high density of CC3-positive punctae was observed in the three lobes (Fig. 2c). Additional examples for all three strains, arranged by increasing CC3 levels in the lateral lobes, are shown in Supplementary Fig. 4a-c. Supplementary Table 1 shows a quantitative summary of the architectural phenotypes from salivary glands of WT and Sg1 transgenic mosquitoes, including those in Fig. 6b, c and Supplementary Fig. 6. The salivary gland morphology of day 1 post-emergence huPAI-1 Sg1 (Supplementary Fig. 4di) is consistent with our previous account of day 1 WT *Anopheles* salivary gland architecture^38^, and levels of CC3 in the lateral lobes are low and diffuse at this early time point. These results suggest that the constitutive expression of huPAI-1 in the salivary glands induces progressive toxicity to the lateral lobes. Consistent with AAPP-driven QF2 expression specifically in female salivary glands, we never observed appreciable CC3 staining in the salivary glands of Sg1 males at day 1 post-emergence (Supplementary Fig. 4dii). Altogether, these experiments indicate that the salivary glands of the huPAI-1 Sg1 transgenic mosquitoes show DL lobe lumen and secretory cavity loss, cell misorganization, loss of cell body adherence to the basement membrane and increased staining for an apoptotic marker.

### Expression of huPAI-1 is not detrimental to mosquito fitness

**Fig. 3:**
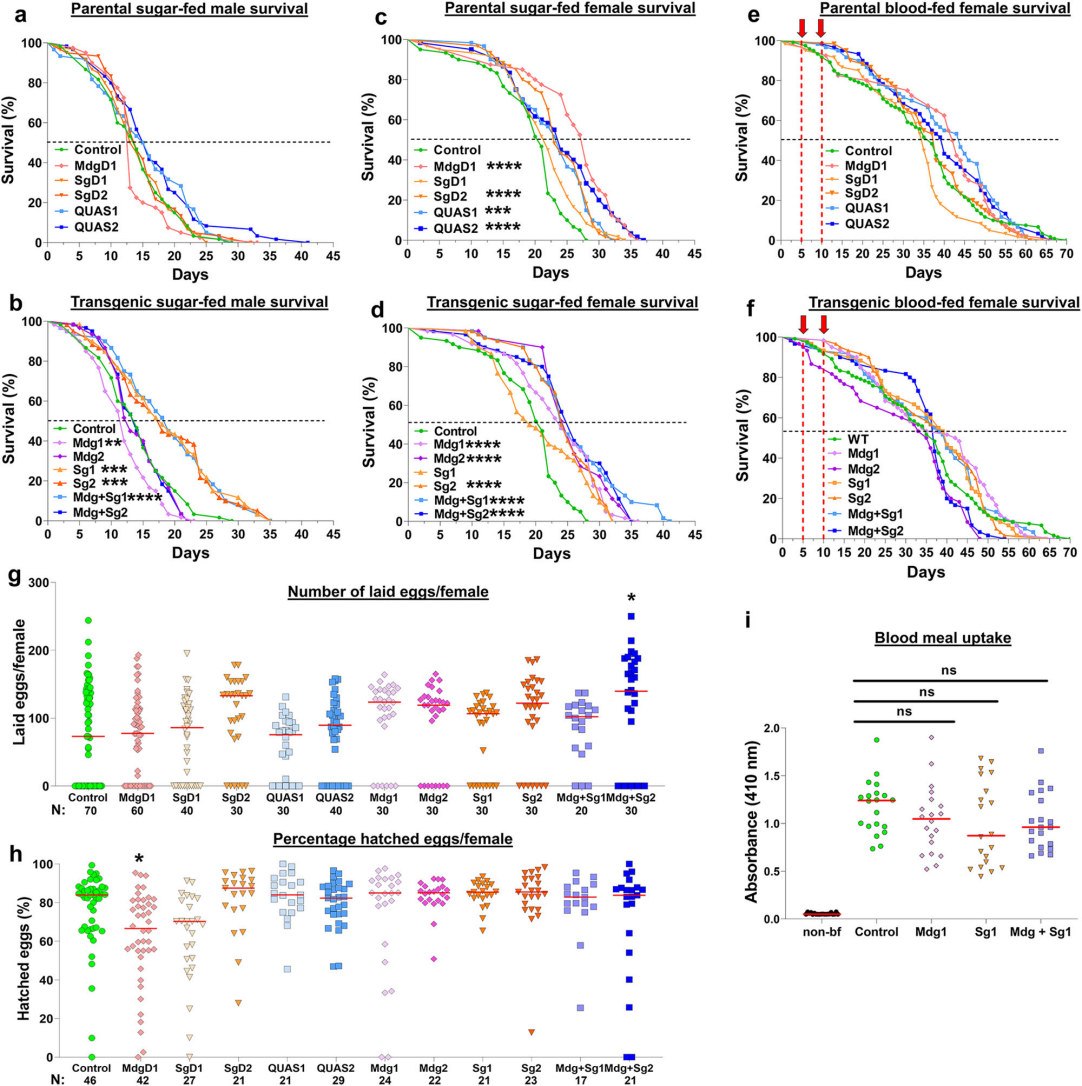
Fitness analysis of *An. stephensi* transgenic lines. **a**–**d** The survival rates for WT, parental and transgenic lines maintained on a sugar meal were evaluated for males (**a**, **b**) and females (**c**, **d**). **e**, **f** Survival was also evaluated for females that received two consecutive blood meals (red arrows). Survival rate was calculated by Kaplan–Meier survival curves, and multiple comparisons were done by two-sided Log-rank test with Bonferroni correction across all lines. **b** **P = 0.0080, ***P = 0.0001, ****P < 0.0001. **c** ***P = 0.0001, ****P < 0.0001. **d** ****P < 0.0001. *N* = 60 mosquitoes for each of three individual biological replicates. **g**, **h** Fecundity (number of laid eggs) (**g**) and fertility (proportion of the laid eggs that hatched) (**h**) of parental and huPAI-1 transgenic lines were evaluated from the same cohort of mosquitoes. Transgenics were compared to WT by Kruskal–Wallis multiple comparisons with Dunn’s post-test. *P = 0.0373. Data from at least three individual biological replicates, except for line Mdg + Sg1 which was tested in duplicate experiments (*N* = 10 females/replicate). **i** Blood uptake is not affected in transgenic mosquitoes expressing huPAI-1. Quantification of protein-bound heme at 410 nm from midguts of WT and transgenic mosquitoes before (non-bf) and after a blood meal. Horizontal red lines represent the median of data pooled from two independent experiments (*N* = 10 females/replicate). Statistical analysis was determined by Kruskal–Wallis multiple comparisons with Dunn’s post-test. ns not significant. *N* = 20 females per experiment.

To determine whether transgene integration or huPAI-1 expression causes a fitness cost to mosquitoes, we analyzed the survival of WT, parental, and huPAI-1 transgenic mosquitoes. Survival curves for sugar-fed males showed no difference between WT and parental (Fig. 3a), whereas all the huPAI-1 expressing lines, except for Mdg2 and Mdg + Sg2, showed a significant increase in survival (Fig. 3b). The comparisons among sugar-fed females showed that the parental and the transgenic lines, except for SgD1 and Sg1, had an increase in survival of 3–7 days when compared to WT at the median time to death (Fig. 3c–d). However, this increased survival was no longer seen when the parental and huPAI-1 transgenic lines were fed on blood, showing a similar survival rate to WT (Fig. 3e–f). In summary, the survival evaluation showed no detrimental effect on the parental or huPAI-1 transgenics.

Next, we estimated the fecundity (number of laid eggs) and fertility (percent hatched eggs) of WT, parental, and huPAI-1 transgenic lines after a single blood meal. The parental or huPAI-1 transgenic lines showed no difference in fecundity when compared to the WT mosquitoes, except for females of the Mdg + Sg2 line that laid significantly more eggs than WT (Fig. 3g). Next, we analyzed the fertility rate from the same cohort of eggs analyzed in the fecundity experiment. All the transgenic lines, parental and huPAI-1, showed similar fertility to WT mosquitoes, except for line MdgD1 which showed a lower fertility rate (Fig. 3h). Based on our results, there is no fitness cost in offspring production for the transgenic lines when compared to the control group.

To determine if huPAI-1 expression in midgut and/or salivary glands affects blood ingestion, we quantified protein-bound heme content in midguts of blood fed females immediately after the blood meal in a subset of transgenic lines. No differences were detected (Fig. 3i), suggesting that the transgenes do not affect blood ingestion. Collectively, our data show that huPAI-1 expression does not impose a fitness cost on the mosquito for the analyzed parameters.

### huPAI-1 reduces mosquito midgut infection by *P. berghei*, *P. falciparum*, and *P. vivax*

**Fig. 4:**
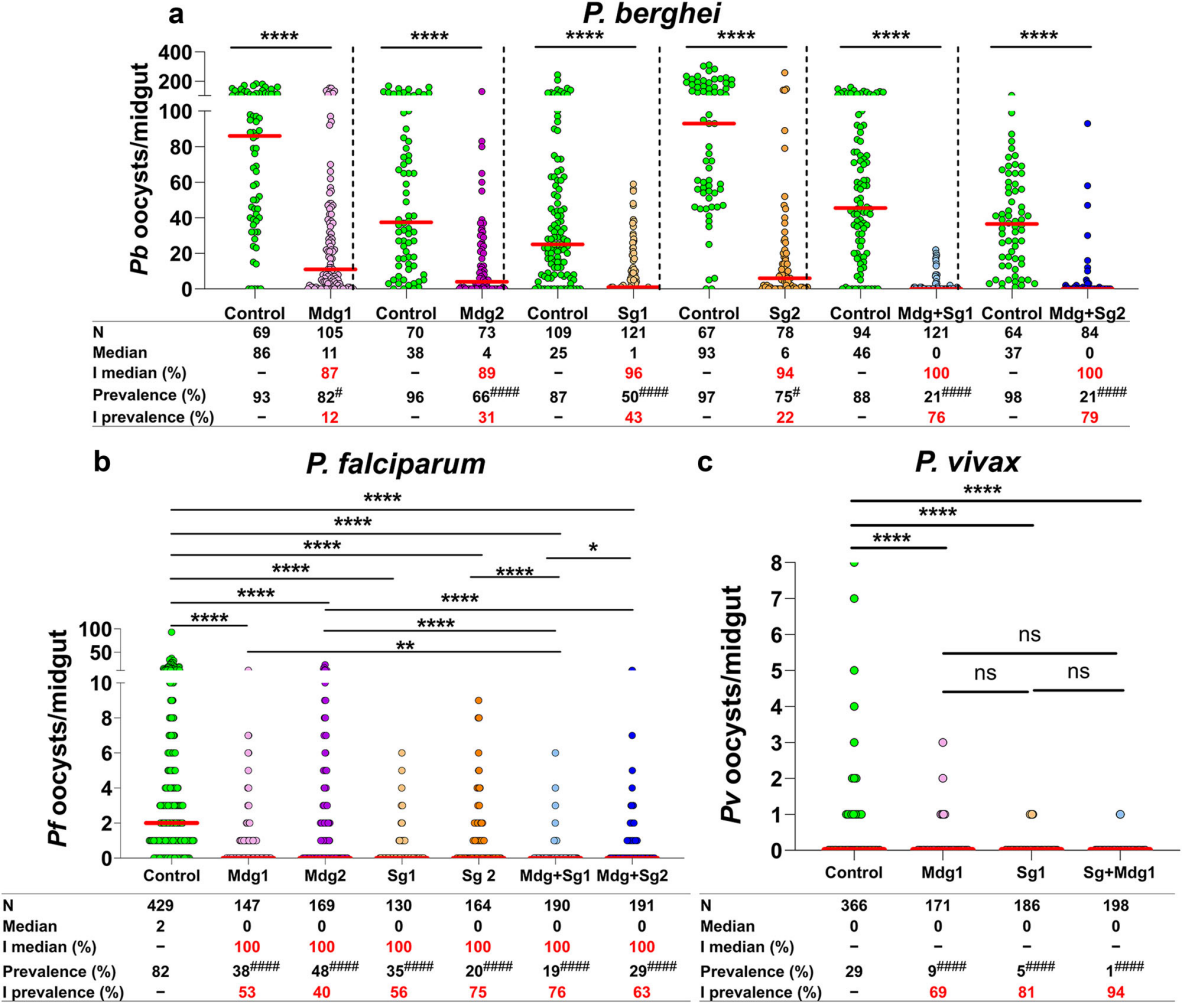
huPAI-1 expression strongly inhibits *P. berghei*, *P. falciparum*, and *P. vivax* oocyst formation. Oocyst numbers were determined in transgenic and control mosquitoes infected with *P. berghei* after feeding on infected mice (**a**), *P. falciparum* by standard membrane feeding assay (SMFA) (**b**), and *P. vivax* by direct feeding on infected monkeys (**c**). Control experiments were done with either WT mosquitoes or the parental lines SgD1, SgD2, or QUAS2 (Supplementary Data 1, Datasets 3–5). *P. falciparum* SMFAs were performed with gametocyte-infected RBCs supplemented with plasma. Horizontal red lines represent the median oocyst number of data pooled from at least three independent experiments shown in Supplementary Data 1, Datasets 3–5. Statistical analysis for oocyst numbers was done by two-tailed Mann–Whitney *U* test For *P. berghei* (**a**), and Kruskal–Wallis multiple comparisons with Dunn’s posttest for *P. falciparum* (**b**) and *P. vivax* (**c**), *P < 0.0477, **P < 0.0016, ****P < 0.0001. Statistical analysis for prevalence was done by Fisher’s exact test for *P. berghei* (^#^*P* < 0.05, ^####^*P* < 0.0001) or Chi-squared test for *P. falciparum* (*χ*^2^ = 350.4, d.f. = 6, ^####^*P* < 0.0001) and *P. vivax* (*χ*^2^ = 12.10, d.f. = 2, *P* = 0.0024). Individual comparisons for *P. falciparum* and *P. vivax* prevalence shown in tables (**b**, **c**): ^####^*P* < 0.0001. I % inhibition, N number of analyzed mosquitoes. The percentage inhibition of median and prevalence was calculated as follows: 100 × [(number of oocysts in the control − number of oocysts in the experimental)/(number of oocysts in the control)].

To assess the effect of huPAI-1 expression on *Plasmodium* midgut infection, we first performed transmission-blocking assays with the rodent malaria parasite *P. berghei*. In each assay, a single *P. berghei*-infected mouse was used to simultaneously feed a control (either WT or the parental lines) group and an individual huPAI-1 transgenic line. Hence, on average, control and transgenic mosquitoes ingest the same number of parasites. Mosquito midguts were dissected at 12 days post-feeding and the number of oocysts per midgut was determined by microscopy. A strong inhibition of oocyst formation was observed for all transgenic lines expressing huPAI-1 and inhibition was strongest in mosquitoes that express huPAI-1 in both midgut and salivary glands (Fig. 4a and Supplementary Data 1, Dataset 3). Salivary gland huPAI-1 transgenics strongly inhibited oocyst formation in the midgut, which is not surprising, since mosquitoes ingest saliva during blood feeding^35^, and saliva huPAI-1 can be detected in the midgut of Sg transgenics (Fig. 1d, g).

To determine if oocyst formation is affected by the site or number of transgene insertions, or by the expression of the QF2 transcription factor, we performed *P. berghei* transmission-blocking assays with the parental driver and effector lines. No difference was observed in the oocyst median, or prevalence between the WT and each of the parental lines (Supplementary Fig. 5a, Supplementary Data 1, Dataset 3). These data show that the reduction in *P. berghei* oocyst formation in mosquitoes expressing huPAI-1 is not due to the transgene insertion site, the number of plasmid insertions or expression of the QF2/QUAS system but is a result of midgut and salivary gland huPAI-1 expression.

Next, we performed SMFAs to evaluate *P. falciparum* NF54 midgut infection. Expression of huPAI-1 in the midgut and/or the salivary glands significantly reduced oocyst numbers and the prevalence of infection in the transgenic lines when compared to WT (Fig. 4b and Supplementary Data 1, Dataset 4). As seen in *P. berghei*, we observed a stronger inhibition of *P. falciparum* oocyst formation in transgenic mosquitoes expressing huPAI-1 in both midgut and salivary glands. We then performed transmission-blocking assays for *P. vivax* (Chesson strain) using nonhuman primates (NHP) *Saimiri boliviensis* as hosts. WT and transgenic mosquitoes were simultaneously fed on the same gametocyte-positive primate, and mosquito midguts were dissected seven days post-infection to determine oocyst numbers. We observed a significant reduction in the mean oocyst number and a strong inhibition in infection prevalence in mosquitoes expressing huPAI-1 in either the salivary glands, the midgut, or both tissues (Fig. 4c and Supplementary Data 1, Dataset 5). Altogether, our data show that huPAI-1 expression strongly inhibits oocyst formation of three *Plasmodium* species, suggesting that parasite utilization of the mammalian fibrinolytic system for mosquito infection is conserved across *Plasmodium* parasites.

### Plasmin supplementation restores midgut infection of transgenic mosquitoes

**Fig. 5:**
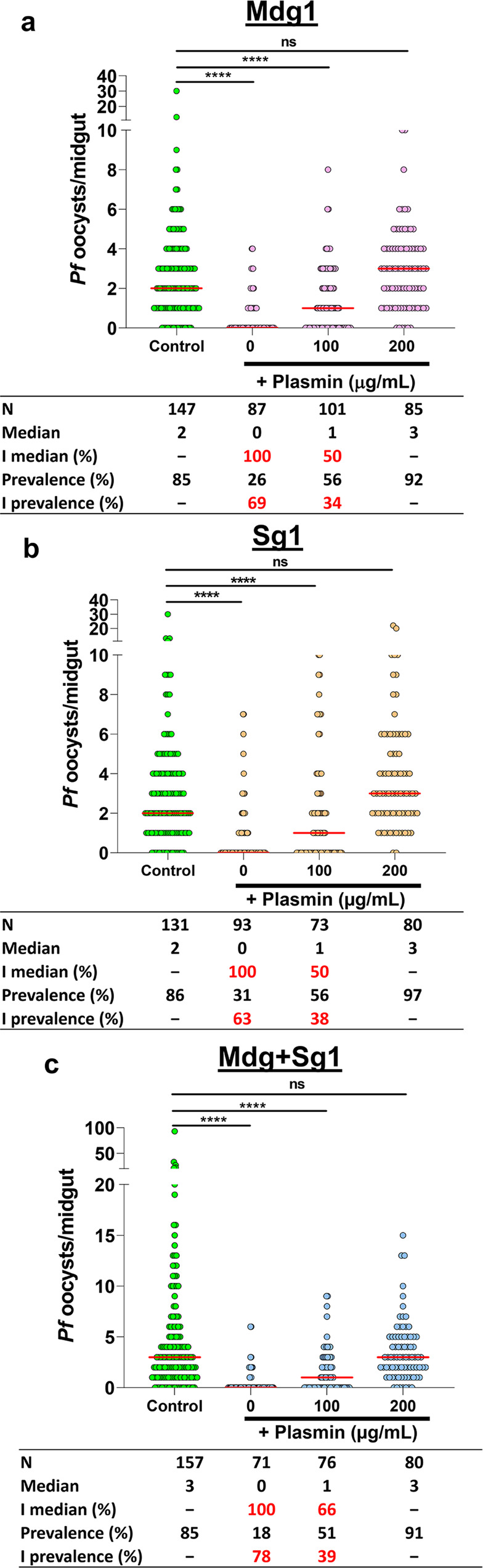
*P. falciparum* midgut infection is restored by plasmin supplementation. **a**–**c** WT and transgenic mosquitoes were fed by SMFA with *P. falciparum* infected blood supplemented with plasma plus increasing concentrations of plasmin. Horizontal red lines represent the median oocyst number of data pooled from three independent experiments shown in Supplementary Data 1, Dataset 6. Statistical analysis was done by Kruskal–Wallis multiple comparisons with Dunn’s posttest, ****P < 0.001, ns not significant. I inhibition, N number of analyzed mosquitoes. The percent inhibition of median, and prevalence was calculated as follows: 100 × [(number of oocysts in the control − number of oocysts in the experimental)/(number of oocysts in the control)].

To determine if the inhibitory effect of huPAI-1 on oocyst formation was due to the specific inhibition of plasminogen activation by tPA and uPA, we performed SMFAs by supplementing *P. falciparum* infectious blood with increasing concentrations of plasmin (PAI-1 inhibits tPA and uPA, but not plasmin activity). Supplementation of infectious blood with 100 μg/mL plasmin partially reversed the inhibition by mosquito-expressed huPAI-1 whereas infection was fully rescued in all transgenics by supplementing the infectious blood meal with 200 μg/mL of plasmin (Fig. 5 and Supplementary Data 1, Dataset 6). The concentrations of plasmin used in these experiments were chosen based on the physiological concentrations of plasminogen and α2-antiplasmin, the inhibitor of plasmin, in the blood (~200 μg/mL). We conclude that inhibition of oocyst formation by huPAI-1 in transgenic mosquitoes is via specific inhibition of plasminogen activation, supporting our previous reports showing that *Plasmodium* parasites hijack the mammalian fibrinolytic system to infect the mosquito^6,7^.

### Salivary gland infection is inhibited in huPAI-1 transgenic mosquitoes

**Fig. 6:**
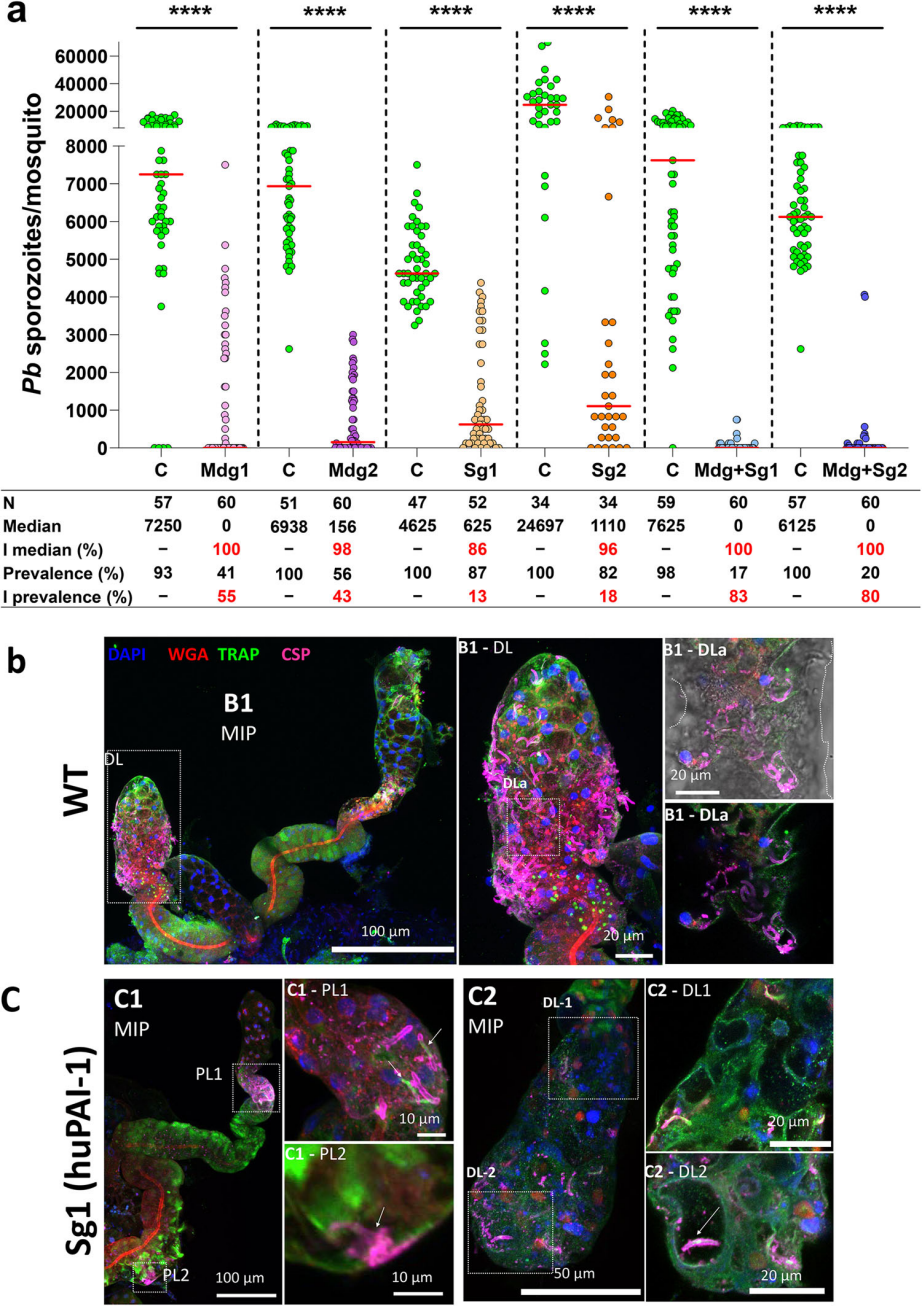
Salivary gland infection is inhibited in transgenic mosquitoes. **a** huPAI-1 reduces sporozoite numbers in the salivary glands of transgenic mosquitoes. Control and transgenic mosquitoes were simultaneously fed on the same *P. berghei* infected mouse and salivary gland sporozoite numbers were determined 21 days post-feeding. Control experiments were done with either WT mosquitoes or the parental lines MdgD1, QUAS1, QUAS2, SgD1 (Supplementary Data 1, Dataset 7). Horizontal red lines represent the median sporozoite number of data pooled from three independent experiments shown in Supplementary Data 1. Control groups were done with either WT mosquitoes or the parental lines SgD1, MdgD1, QUAS1 or QUAS2 (Supplementary Fig. 5 and Supplementary Data 1, Dataset 7). Statistical analysis was done by two-tailed Mann–Whitney U test, ****P < 0.0001. C control, I inhibition, N number of analyzed mosquitoes. The percent inhibition of median and prevalence was calculated as follows: 100 × [(number of sporozoites in the control − number of sporozoites in the experimental)/(number of sporozoites in the control)]. **b**, **c** Salivary glands expressing huPAI-1 are poorly invaded by sporozoites. Salivary glands dissected 21 days post infection were stained with anti-TRAP (parasite; green) and anti-CSP (parasite; magenta) antisera, DAPI (DNA, blue), and WGA (chitin/O-GlcNAcylation; red). Representative WT and Sg1 (**c**) salivary gland 3D projection (MIP) or single slice confocal images are shown. **b** WT salivary glands show robust invasion, including sporozoite occupancy of secretory cavities and lumens. **c** Salivary glands of mosquitoes overexpressing huPAI-1 show sparse invasion frequently mistargeted to the PL lobes (c1-PL1) and dead parasites associated with the basement membrane (c1-PL2, arrow). Interestingly, loss of most sporozoite CSP staining is observed upon invasion (c1-PL1, arrows). Rare DL lobes (4/33) that possessed secretory cavities and/or a lumen showed sporozoite invasion of these regions (arrow in c2-DL2). Data presented in panels b-c are representative of at least two independent experiments. Additional examples of infected salivary glands from the Sg1 line are shown in Supplementary Fig. 6.

We showed that *P. berghei* midgut infection is reduced in huPAI-1 transgenic mosquito lines. However, a significant number of oocysts still developed, especially in the Mdg and Sg lines (Fig. 4a). These oocysts can produce sporozoites that will infect the salivary glands and could be transmitted to the mammalian host during the next blood feeding. We found a significant reduction in salivary gland sporozoite numbers and prevalence in both Sg and Mg lines, while inhibition was stronger in the Mdg + Sg lines (Fig. 6a and Supplementary data 1, Dataset 7). No difference was observed in salivary gland infection between the WT mosquitoes and each of the parental lines (Supplementary Fig. 5b and Supplementary Data 1, Dataset 7). These data show the efficacy of huPAI-1 expression in reducing the salivary gland infection.

Expression of PAI-1 in the salivary glands induced significant structural damage to the distal part of the salivary gland lateral lobes (Fig. 2 and Supplementary Fig. 4). We next investigated if the structural changes caused by salivary gland expression of huPAI-1 led to differences in *P. berghei* sporozoite invasion. We performed IFAs of salivary glands dissected 21 days post infection using antibodies to detect the sporozoite surface proteins TRAP (green) and CSP (magenta), as well as DAPI and WGA to stain the DNA (blue), and O-GlcNAcylation/Chitin (red), respectively (Fig. 6b, c and Supplementary Fig. 6). In WT *An. stephensi*, sporozoites were found in high numbers both at the basement membrane and within DL lobes (Fig. 6b) confirming our observations from a previous study^41^. Staining for TRAP and CSP showed that sporozoites had moved through the salivary gland and into the cell secretory cavities and the gland DL lumen (Fig. 6b1- DLa). Sporozoites at the basal membrane show a strong staining for TRAP and CSP, whereas invading parasites lose most of the CSP signal. In the salivary glands of Sg1, far fewer sporozoites were observed in association with the basement membrane and/or occupying the interior of the salivary gland (Fig. 6c1, c2i, and Supplementary Fig. 6)^41^. Additional examples of infected Sg1 salivary glands are shown in Supplementary Fig. 6. Interestingly, in the sparse infection of Sg1 salivary glands, we observed invasion attempts in areas beyond the DL lobes, where the vast majority of sporozoite invasions of WT salivary glands occur. One case in the PL lobe, showed multiple parasites external to the basement membrane (strong CSP signal), and several that had invaded (strong TRAP signal and loss of most CSP signal; Fig. 6c1-PL1). Another PL lobe invasion event was unsuccessful, and only misshapen sporozoite remnants remained (Fig. 6c1-PL2). Rare M lobe invasion events were also observed (Supplementary Fig. 6; cyan arrows). In rare Sg1 DL lobes that retained secretory cavities and/or a reduced central lumen (4 DL lobes of 33 inspected), individual or bundled sporozoites were usually observed (Fig. 6c2 and Supplementary Fig. 6a). In contrast, most WT DL lobes contained secretory cavity or lumenal sporozoites (Fig. 6b)^41^. Some DL lobes from infected Sg1 contained abundant TRAP staining, but few sporozoites (Supplementary Fig. 6b, b’), suggesting some parasites may die or degrade within the salivary glands. A summary of the architecture and sporozoite invasion of WT and transgenic salivary glands is shown in Supplementary Table 1. No differences in oocyst diameter were detected in parasites developing in WT, Mdg1 or Mdg + Sg1 transgenic mosquitoes 12 days post infection (Supplementary Fig. 7), showing that oocysts develop at the same rate and suggesting that sporozoite formation is not affected in transgenic mosquitoes. Altogether, these data show that huPAI-1 expressing salivary glands are more poorly invaded by *Plasmodium* sporozoites than WT salivary glands, possibly as a result of increased apoptosis and tissue deformation of the DLs.

### Malaria transmission is impaired in huPAI-1 transgenic mosquitoes

**Fig. 7:**
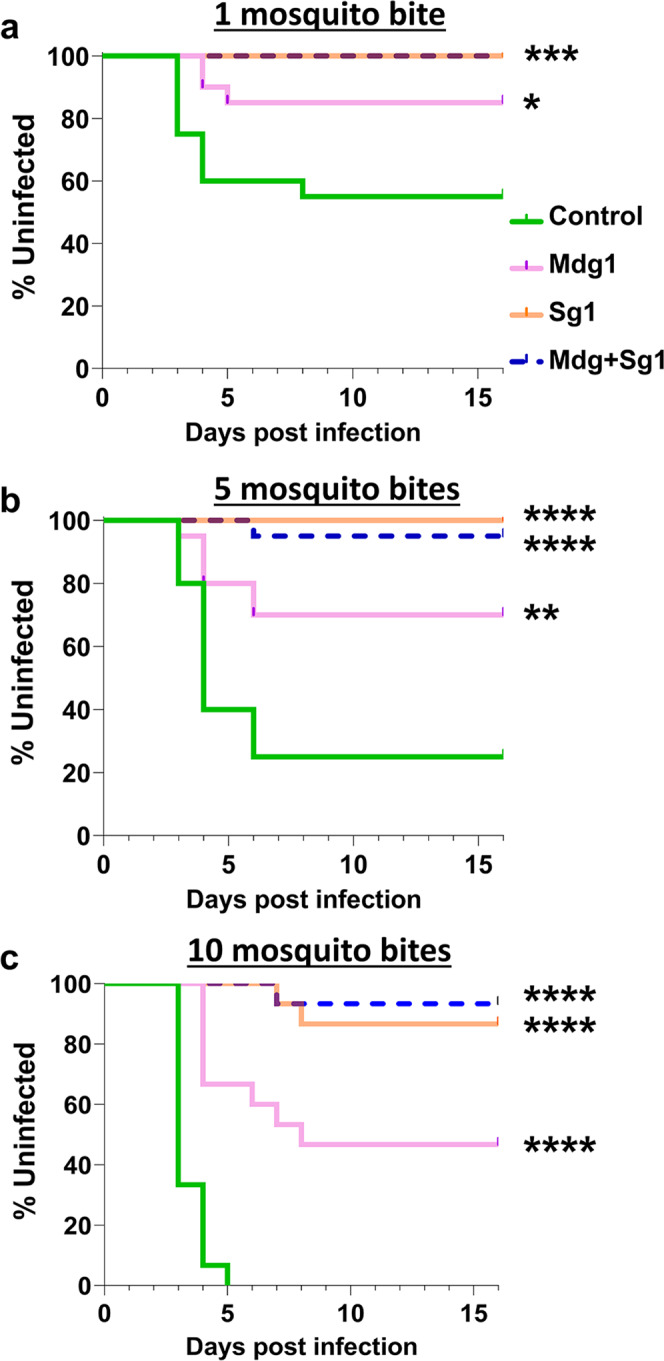
Transgenic mosquitoes strongly inhibit *P. berghei* transmission to mice. **a**–**c** WT and transgenic mosquitoes were fed on the same *P. berghei* infected mouse, and unfed mosquitoes were removed. To determine their potential to transmit malaria, mosquitoes were randomly selected at 21 days post-infection and fed on naïve mice (challenge). Each mouse was challenged with one (**a**), five (**b**), or ten (**c**) mosquitoes. Infection was determined by daily monitoring blood-stage parasites on Giemsa-stained blood smears. Statistical analysis was determined by Log-rank (Mantel-Cox) test with *α* = 0.05 and *k* = 0.0166. Data from 1 and 5 mosquitoes, *N* = 10 mice per group per replicate, with two replicates (total 20 mice). Data from 10 mosquitoes, *N* = 15 mice, 10 mice per group for one replicate and 5 mice per group for the second replicate (total 15 mice). **a**: *P = 0.0306, ***P = 0.0007. **b**: **P = 0.0036, ****P < 0.0001. **c**: ****P < 0.0001. The oocyst number for the mosquitoes used for the challenge is shown in Supplementary Data 1, Dataset 8.

Our data shows that *Plasmodium* infection in mosquitoes is reduced in the midgut and salivary glands of the huPAI-1 transgenic lines. However, even when sporozoite numbers are significantly reduced in salivary glands, only a few sporozoites delivered into the dermis of a naïve host are required to initiate an infection. To determine the effect of huPAI-1 transgenics on malaria transmission, we challenged mice with the bite of WT or transgenic mosquitoes that had ingested an infectious blood meal. WT and transgenic mosquitoes were fed on the same *P. berghei* infected mouse and at 21 days post-feeding, one, five, or ten randomly selected mosquitoes were blood-fed on a non-infected mouse. For each line, mosquito infectivity was confirmed by counting the number of oocysts. As shown earlier, a significant reduction in the infection intensity and prevalence was observed for Mdg1, Sg1, and Mdg + Sg1 lines (Supplementary Data 1). When mice were challenged with the bite of one WT mosquito per mouse, 45% of the mice became infected (Fig. 7a), while challenging with transgenic lines resulted in significant protection: 85% for Mdg1 and 100% for the Sg1 and the Mdg + Sg1 lines (Fig. 7a). When mice were challenged with five mosquitoes, 75% of the mice bitten by WT mosquitoes became infected, whereas the transgenic lines showed significant protection: 70% for Mdg1, 100% for Sg1, and 95% for Mdg + Sg1 (Fig. 7b). Finally, challenging with 10 WT mosquitoes resulted in 100% of mice becoming infected with malaria, whereas challenging with 10 transgenic mosquitoes resulted in the protection of 46% for Mdg1, 86% for Sg1, and 93% protection for Mdg + Sg1 (Fig. 7c).

In summary, our data show that huPAI-1 expression in mosquito midguts and/or salivary glands strongly reduces *Plasmodium* parasite development in the mosquito and greatly impairs malaria transmission to a new host.

## Discussion

Our previous work showed the importance of the fibrinolytic system in facilitating *Plasmodium* infection of the mosquito and the vertebrate host^6,7^. These studies suggested that targeting plasminogen activation could serve a strategy for blocking malaria transmission. Here, we show a proof-of-concept approach where transgenic expression of huPAI-1, an inhibitor of plasminogen activation, by *An. stephensi* mosquitoes strongly inhibits *Plasmodium* infection of the mosquito and transmission to the mammalian host.

To our knowledge, our study is the first to report transgenic mosquitoes expressing a human protein with potent anti-plasmodial activity. Previous studies reported the development of refractory transgenic *Anopheles* mosquitoes expressing anti-plasmodial molecules in the midgut or the hemolymph. These include midgut expression of single-chain antibodies against parasite surface proteins^25^, mosquito miRNAs and regulators of the mosquito immune system^18,19^, peptides that block parasite interaction with the midgut epithelium^21^, and antimicrobial peptides and toxins that kill the parasite^20,22–24,42^. In addition, transgenic expression of the mouse apoptotic factor Bcl-2-associated X protein (Bax) in salivary glands reduced parasite infectivity to mosquitoes^43^. Several of these transgenics achieved potent inhibition of parasite transmission and are currently being pursued as interventions to block malaria transmission. However, targeting the parasite may impose selective pressure for the development of evasion mechanisms against the transgene, and targeting mosquito genes may impose a fitness cost that could compromise the competitiveness of the mosquito relative to the wild population. Our data show that expression of huPAI-1 is not detrimental to the mosquito fitness and can increase mosquito survival when feeding on sugar. Different from previous approaches, our strategy does not directly target the parasite or the mosquito. Instead, we inhibit the activation of plasminogen at the parasite surface, thus preventing the parasite from using plasmin for mosquito infection. This approach limits the selective pressure that a transgene can exert on the parasite, thereby preventing the development of evasion mechanisms against the effector molecule. Furthermore, the expression of effector molecules in the mosquito saliva allows to target both, the sporozoite in the salivary gland or the skin, and the midgut stages of the parasite. However, further experiments in animal models will need to be performed to study immune or inflammatory responses against the effector molecule injected by the mosquito. In addition, by using the binary QF2/QUAS system^27,44^ we achieved strong expression of the transgene, and we generated new midgut and salivary gland QF2 driver lines which can be used to constitutively express the protein of choice in one, or both, of these tissues.

The transgenic lines show tissue-specific expression of huPAI-1 and secretion into the midgut lumen or in the saliva. Expression of QF2 or huPAI-1 in the midgut did not cause any structural changes to the tissue, whereas QF2 expression in the salivary glands resulted in slight toxicity. QF2 toxicity was previously reported when the Q-binary system was adapted for expression in *Drosophila*^40,44^. However, expression of huPAI-1 in salivary glands induced a strong toxicity with increased cell apoptosis, detachment of the glandular cells from the basal lamina, lack of cell polarization, and no organizational pattern between the cells of the distal lateral lobes. This toxicity is observed early in adult development, suggesting that huPAI-1, a serpin, could be targeting one or more salivary gland serine proteases^45^. When analyzed for fitness, neither the expression of QF2 nor the expression of huPAI-1 affected mosquito longevity, blood meal uptake or offspring production under our laboratory conditions.

Expression of huPAI-1 in the midgut and salivary glands resulted in strong inhibition of *P. berghei*, *P. falciparum*, and *P. vivax* oocyst formation, indicating that co-opting the mammalian fibrinolytic system for infection of the mosquito is a conserved mechanism used by *Plasmodium* parasites^6,7^. Interestingly, huPAI-1 secreted in the saliva is ingested during feeding and can be detected in the mosquito midgut lumen, where it strongly blocks oocyst development. These results confirm previous reports that mosquitoes ingest a considerable amount of saliva during blood feeding^35,46,47^. Oocyst inhibition was stronger in mosquitoes expressing huPAI-1 in the saliva than those expressing it in the midgut. The stronger inhibition by saliva huPAI-1 could be explained by differences in protein secretion between the salivary gland and midgut cells. Saliva proteins are constitutively secreted into the secretory cavities of the salivary gland cells^48,49^. During blood feeding, saliva is ingested together with the blood facilitating the dissemination of huPAI-1 throughout the blood bolus and access to tPA and uPA. In contrast, most proteins secreted into the midgut lumen are stored in apical vesicles that are released into the midgut lumen during physical distention caused by blood meal ingestion^50–53^. Interestingly, we detected huPAI-1 expression in punctae structures that resemble vesicles in the midgut which suggest that huPAI-1 could also be secreted upon blood ingestion. This type of secretion could slow down the effect of midgut-secreted huPAI-1 since the protein must diffuse inward into the blood bolus to gain access to tPA and uPA. Furthermore, some of the parental lines used in this study contain multiple transgene insertions (up to 3 insertions) which could enhance huPAI-1 expression and induce a more potent inhibition of parasite development, but also increase the chances of disrupting a locus essential for mosquito fitness. Although our results do not show any fitness cost in transgenic mosquitoes, future experiments could evaluate fitness and parasite inhibition by huPAI-1 in mosquitoes with single-copy integrations generated through backcrossing with WT mosquitoes and isolation of isogenic lines.

The sporozoite number in the salivary glands of transgenic mosquitoes was also significantly reduced which can be explained by the reduction in oocyst numbers. However, mosquitoes expressing huPAI-1 in the saliva were significantly less likely to transmit malaria than mosquitoes expressing huPAI-1 only in the midgut, although both groups had comparable sporozoite numbers in the salivary glands. Since the distal lateral lobes are the preferential site of sporozoite invasion^41,54–56^, damage to this region induced by huPAI-1 expression could potentially impose an additional physical barrier to sporozoite invasion. For example, the distal lobes of the SgD lines, which were readily invaded by sporozoites, had a moderate degree of cell misorganization with low levels of apoptosis and detachment from the basement membrane, whereas distal lobes expressing huPAI-1 showed high levels of apoptosis, significant structural defects and were poorly invaded. IFAs show that the majority of sporozoites that invade salivary glands expressing huPAI-1 remained trapped within the cytoplasm of the salivary gland cells, thereby preventing their migration to the secretory cavity from where they are transmitted to a new host during subsequent blood feedings. This is in agreement with previous studies showing that variations in the salivary gland architecture can affect sporozoite invasion, imposing a bottleneck for transmission^2,39,41,49^. In addition, PAI-1 inhibits plasmin formation on the sporozoite surface which is required for migration through the extracellular matrix of the dermis^6^. It is possible that the huPAI-1 deposited with the saliva at the bite site could also reduce sporozoite migration in the dermis, further reducing malaria transmission by the salivary gland transgenics. This hypothesis could not be tested due to the low number of sporozoites detected in the transgenic salivary glands.

In summary, the expression of huPAI-1 in the saliva and the midgut of *An. stephensi* mosquitoes impose additional barriers to parasite development and reduce the number of infectious sporozoites that can be transmitted which is an important determinant of malaria transmission^57^. This report validates the possibility of targeting the fibrinolytic system using genetically engineered mosquitoes to prevent the transmission of multiple malaria parasite species.

## Methods

### Animal handling and ethics protocol

All animal procedures were performed in strict accordance with the National Institutes of Health (NIH) Guidelines under protocols approved by the National Institute of Allergy and Infectious Diseases Animal Care and Use Committees (NIAID ACUC) and from Bioqual Inc. (Rockville, MD, USA). *Saimiri boliviensis* were obtained from NIH-approved sources and housed in compliance with the Animal Welfare Act and the Guide for the Care and Use of Laboratory Animals^58^. The studies were done following the approved animal study proposals LMVR-22 and LMVR-9. Mosquito infections were done according to the guidelines of the LMVR Insectary SOPs: 203, 601, 604, 605, and 606. Commercial anonymous human blood was used for parasite cultures and mosquito feeding, and informed consent was therefore not applicable. Human blood for *P. falciparum* culturing was collected from healthy volunteers. All individuals gave written informed consent and enrolled in a protocol approved by the National Institutes of Health Clinical Center Institutional Review Board (NIH protocol 99-CC-0168 “*Collection and Distribution of Blood Components from Healthy Donors for* In Vitro *Use*”).

### Mosquito rearing and parasite culture

*Anopheles stephensi* Liston strain (Feldmann et al., 1989) and *An. stephensi* transgenic lines were reared according to LMVR Insectary procedures (LMVR, NIAID, NIH; Rockville, MD, USA) at 27 °C and 80% relative humidity, with a 12 h/12 h light/dark cycle. Larvae were reared in trays with dechlorinated water and fed on cat food (Cat Chow^®^; Purina), while adults were maintained with cotton pads soaked in 10% corn syrup solution (Karo^®^, ACH Food Companies). For colony maintenance and egg production, the females were fed with bovine blood (Lampire Biological Laboratories) using artificial membrane feeders. For fitness evaluation, the mosquitoes were fed on Swiss Webster mice.

*P. falciparum* NF54 gametocytes were produced according to Canepa et al.^59^. Briefly, the parasites were maintained in O + human erythrocytes using RPMI 1640 medium supplemented with 25 mM HEPES, 50 mg/l hypoxanthine, 25 mM NaHCO3, and 10% (v/v) heat-inactivated type O + human serum (Interstate Blood Bank, Inc.) at 37 °C and with a gas mixture of 5% O_2_, 5% CO_2_, and balanced N_2_. For feeding, 14–17-day old mature gametocytes were pelleted by centrifugation (3 min, 2500 g), resuspended with O + human RBC to 0.15–0.2% gametocytaemia and diluted to 40% hematocrit with human serum. All manipulations were done maintaining the cultures, tubes, and feeders at 37 °C.

### Plasmid constructs

The pXL-BACII-ECFP-15XQUAS_TATA-HuPAI1-SV40 containing the huPAI-1 expression cassette and the ECFP under the eye-specific promoter 3xP3 was used to generate the parental QUAS-huPAI-1 effector lines. The coding sequence for the human PAI-1 gene (NP_000593.1) was synthesized (Genescript) to include the stabilizing mutations N150H, K154T, Q319L, and M354I. These mutations increase the half-life of PAI-1 from 2 h to 147 h at 37 °C^32^. The PAI-1 coding sequence was amplified using primers PAI-FW and PAI-RV (Table S3) and InFusion-cloned into plasmid pXL-BACII-ECFP-15XQUAS_TATA-SV40^27^ previously linearized with XhoI.

The pXL-BACII-DsRed-AsAper-QF2-hsp70 containing the QF2 transcription factor under the control of the midgut-specific AsAper promoter and the DsRed marker under the eye-specific promoter 3xP3 was used to generate the parental Mg-QF2 driver line. The AsAper promoter (1.5 kb) was PCR amplified from *An. stephensi* gDNA with primers MgP-FW and MgP-RV (Table S3). The PCR product was InFusion-cloned into plasmid pXL-BACII-DsRed-QF2-hsp70^27^ previously linearized with XhoI.

The pXL-BACII-YFP-AsAAPP-QF2-hsp70 containing the QF2 transcription factor under the control of the midgut-specific AsAAP promoter and the YFP marker under the eye-specific promoter 3xP3, was used to generate the parental Sg-QF2 driver lines. The YFP coding sequence was amplified from plasmid pBM2-YFP using primers YFP-FW and YFP-RV (Table S3). The PCR product was InFusion-cloned into plasmid pXL-BACII-DsRed-QF2-hsp70 previously digested with ApaI and NotI to produce plasmid pXL-BACII-YFP-QF2-hsp70. The AsAAPP promoter consisting of a 1.7 kb upstream of the start codon^29^ was PCR amplified from *An. stephensi* gDNA using primers SgP-FW and SgP-RV (Table S3). The PCR product was InFusion-cloned into plasmid pXL-BACII-YFP-QF2-hsp70 previously linearized with XhoI.

### Generation of transgenic mosquitoes

The plasmid constructs were microinjected together with the helper plasmid phsp-pbac containing the transposase into the embryos of *An. stephensi* (Liston strain) according to Volohonsky et al.^33^. Briefly, transformation plasmids were purified using the EndoFree Maxi Prep Kit (Qiagen) and resuspended in injection buffer (0.1 mM NaHPO4 pH 6.8 and 5 mM KCl) at a concentration of 250 ng/µL for the transformation plasmid and 200 ng/µl for the helper plasmid phsp-pBac containing the transposase^60^. The plasmid mix was injected into *An. stephensi* embryos using a FemtoJet Microinjector (Eppendorf). Integration into the genome was mediated by piggyBac inverted repeated sequences flanking the transgenes in the effector and driver plasmids. Third instar larvae of G_0_ survivors were screened for transient expression of the 3xP3-dsRed marker (red eyes), the 3xP3-YFP marker (yellow eyes), or the 3xP3-CFP marker (blue eyes). Adults obtained from the fluorescent marker screening were crossed to WT mosquitoes to generate independent transgenic lines.

Splinkerette PCR and PCR sequencing were used to determine the transgene insertion sites in the *An. stephensi* genome for each of the parental transgenic lines^34^. To isolate genomic DNA, fourth instar larvae were collected into 100 μL of Lysis Buffer (100 mM Tris-HCl pH 8.0, 0.5% SDS, 50 mM NaCl, 100 mM EDTA) and macerated on ice. Proteinase K (Invitrogen) was added to the lysate at a concentration of 1 mg/mL and the lysate was incubated at 55 °C for 1 h. RNaseA (QIAGEN Puregene Cell Kit; QIAGEN) was added at 0.5 mg/mL, the lysate was incubated at room temperature for 20 min and centrifuged at 10,000 × *g* for 10 min at 4 °C. The aqueous phase was collected from phenol-chloroform-isoamyl organic extraction, followed by ammonium acetate/ethanol precipitation and ethanol washing (Cold Springs Harbor Protocols). DNA concentration was measured by spectrophotometry on a DeNovix DS-11 (DeNovix). One microgram of genomic DNA was incubated with 10 units of BstYI restriction enzyme (New England Biolabs) and 1X NEB Buffer 2.1 (New England Biolabs) in a total volume of 35 µl and incubated overnight at 60 °C. The digested genome was incubated with 1.8 μg of annealed splinkerette oligonucleotide, 1X NEB Buffer, and 400 units of T4 DNA Ligase (New England Biolabs) for 6 h at room temperature. The ligated DNA was used to amplify the regions of interest using nested PCR amplification. Both rounds of amplifications were conducted with 1X Phusion High-Fidelity PCR Master Mix with HF Buffer (Thermo Fisher Scientific). The primers used in these experiments are shown in Table S3.

In the first round of amplification, the splink#1 primer was used in combination with the piggybac LE#1 or piggybac RE#1 primers to amplify both ends (5′ and 3′) of the 10μl (~0.5 µg) of splinkerette-ligated DNA. PCR conditions were: 1 round of 98 °C for 75 s; 2 rounds of 98 °C for 20 s and 64 °C for 15 s; 30 rounds of 98 °C for 20 s, 57 °C for 15 s, and 72 °C for 2 min; and 1 round of 72 C° for 7 min. For the second round of amplification, the splink#2 primer was added in combination with the piggybac LE#2 or piggybac RE#2 primers to the product of the first round of PCR. The PCR conditions for the second amplification were identical to the first round of PCR but excluded the second step, two rounds of 98 °C for 20 s and 64 °C for 15 s. The amplified PCR products were resolved in a 1.5% agarose gel stained with ethidium bromide, and the amplified DNA bands from 5′ and 3′ ends (Supplementary Fig. 2A) were individually excised and purified with QIAquick^®^ Gel Extraction Kit (QIAGEN). Purified PCR products were cloned into 25 ng of pJET1.2/blunt plasmid (Thermo Fisher Scientific) and transformed into NEB 5-alpha Competent *Escherichia coli* (High Efficiency, Thermo Fisher Scientific). Plasmids were isolated from individual colonies and sequenced with the universal primers pJET12F and pJET12R (Eurofins). The sequences were aligned to the *An. stephensi* genome in the library (VectorBase and NCBI BLAST) to identify the location of the transgenes (Supplementary Fig. 2B).

To induce midgut- or salivary gland-specific expression of huPAI-1, QF2 driver lines were randomly crossed to QUAS-huPAI-1 effector lines (Supplementary Fig. 2C). The offspring of each cross was selected by the specific combination of eye fluorescence reporter. To increase the homozygosity, each cross was continuously screened throughout multiple generations and only mosquitoes positive for the expected combination of fluorescent markers were used for the experiments.

### Reverse transcription-polymerase chain reaction (RT-PCR)

Tissue-specific expression of huPAI-1 in *An. stephensi* transgenic lines were evaluated by RT-PCR. Salivary glands, midguts, and carcasses (abdomen without midgut) were dissected from female mosquitoes in PBS (0.1 M, pH 7.8). Tissues were collected in microtubes containing 1 mL of TRIzol® (Invitrogen), homogenized, and stored at −70 °C until RNA extraction. Total RNA was extracted according to TRIzol® manufacturer’s protocol, resuspended in RNAse free water, and treated with RQ1 RNase-Free DNase® (Promega). After RNA quantification using a DeNovix DS-11 spectrophotometer, 1st strand cDNA was synthesized for each sample using Superscript III (Invitrogen) with random hexamers (Invitrogen) and 500 ng of total RNA per sample. cDNA was treated with RNase H (New England Biolabs) for 10 min at 37 °C and stored at −70 °C until use. The cDNA was used as a template in PCR reactions containing the Taq 2X Master Mix (New England Biolabs) and 5 μM of huPAI-1 specific primers (Table S3). Amplification of S7 ribosomal mRNA was used as loading control. PCR conditions were: 1 hot start of 95 °C for 30 s; 35 cycles of denaturation at 95 °C for 30 s, annealing at 56 °C for 30 s, and elongation at 68 °C 30 s; followed by a final extension at 68 °C for 5 min; and 4 °C indefinitely. PCR products were resolved in 1% agarose gels stained with ethidium bromide.

### Western blotting

huPAI-1 protein synthesis in midgut and salivary glands of the transgenic lines was evaluated by Western blotting. A pool of five midguts and ten salivary glands were dissected in PBS (0.1 M, pH 7.8), collected in microtubes containing RIPA Buffer® (Thermo Fisher Scientific), 1% Halt™ Protease Inhibitor Cocktail (Thermo Fisher Scientific), and 0.1 mM PMSF (Sigma-Aldrich). Samples were homogenized and stored at −70 °C. An equivalent of five midguts (~40–50 µg of protein) and five salivary glands (~10 µg of protein) were resolved in a NuPAGE™ 10% Bis-Tris Protein Gel (Invitrogen) under reducing conditions and transferred to a PVDF membrane Invitrogen™ Power Blotter Select Transfer Stacks (Invitrogen). After the transfer, the membrane was washed with TBST 1% (Sigma-Aldrich), incubated with blocking buffer (5% milk powder in TBST 1%) overnight at 4 °C, and probed with a mouse anti-human PAI-1 antibody (BD Biosciences, clone 41/PAI-1, #612024) at a 1:1000 dilution in TBST 1% overnight at 4 °C. The membrane was washed and incubated with a rabbit anti-mouse HRP-linked antibody (Cell Signaling, # 7074) at a 1:10,000 dilution in TBST 1% for 2 h at room temperature. Detection was done with the SuperSignal™ West Dura Extended Duration Substrate Chemiluminescent Substrate (Thermo Fisher Scientific), and imaged using an Azure Imager c600^®^ (Azure Biosystems).

### Detection of huPAI-1 protein secretion

Secretion of huPAI-1 into the midgut lumen was analyzed using a low-melting agarose feeding assay^36^. In this assay, low melting agarose solubilized in bicarbonate-buffered saline solution is fed to mosquitoes. Once ingested, the agarose solidifies inside the midgut and absorbs any protein secreted by the midgut epithelium. WT and transgenic females were fed with a 1% low-melting agarose solution for 15 min. Midguts from five mosquitoes were dissected on ice-cold PBS (0.1 M, pH 7.8) at 30 min after feeding. The midgut agarose bolus was collected and placed in protease inhibitor solution (as described above), homogenized, and stored at −70 °C. Proteins in the homogenate were resolved by SDS-PAGE and transferred to a PVDF membrane. The membrane was probed with a mouse anti-human PAI-1 antibody (1:1000) and developed as described before. An antibody against the midgut secreted protein heme peroxidase (IMPer) (1:2000^37^;) was used as a positive control, and an antibody against the non-secreted midgut eNOS (1:1000, Millipore Sigma #N217) was used as a negative control.

Secretion of the huPAI-1 protein in saliva was evaluated by spit-blot assay. For this assay, WT or transgenic lines expressing huPAI-1 in the salivary glands were allowed to probe for 10 min on a Pierce® Nitrocellulose Membrane (Thermo Fisher Scientific) pre-warmed at 37 °C on a hot plate. Recombinant huPAI-1 was spotted outside the probing areas as a positive control. The membranes were incubated with a mouse anti-human PAI-1 antibody, as described before. Membranes were stripped with Restore™ Western blotting Stripping Buffer (Thermo Fisher Scientific) and probed with an antibody against the saliva protein AAPP used as a positive saliva control (1:1000^61^;).

### Immunofluorescence assays

For midgut analysis, female mosquitoes were fed with a bicarbonate-buffered saline solution by SMFA^36^. Immediately after feeding, mosquito midguts were dissected in 1× PBS and carefully opened with a longitudinal cut to produce a sheet. Tissues were fixed in 4% paraformaldehyde (Sigma-Aldrich), transferred to microtubes, and washed three times with 1× PBS. The samples were blocked in 5% w/v BSA and 0.1% w/v gelatin in PBST (blocking buffer) for 1 h. The midguts were incubated for 2 h with a mouse anti-human PAI-1 antibody (1:1000 in 1% PBST; Invitrogen). The tissues were washed, blocked for 1 h, and incubated with a secondary antibody conjugated to Alexa Fluor 594 dye (1:2500 in PBST 1%; Thermo Fisher Scientific, #A-11005) for 2 h. Midguts were washed and incubated with Alexa Fluor™ 647 Phalloidin (1:40 in 1× PBS; Thermo Fisher Scientific, #A22287) and Hoechst 33342 (1:10,000 in 1× PBS; Invitrogen) solution in 1X PBS for 30 min at room temperature. The midguts were washed in 1X PBS, transferred to Superfrost™ Plus glass slides (Thermo Fisher Scientific) and mounted with ProLong™ Glass Antifade Mountant (Thermo Fisher Scientific). Images were taken on a Leica SP8 DMI 6000 confocal microscope (Leica Microsystems) equipped with a photomultiplier tube/hybrid detector using a 63× oil immersion objective with a zoom factor of 4. Samples were visualized with lasers specific for emission and excitation range depending on the fluorophore used. Images were taken using sequential acquisition and variable z-steps (z-stacks). Image processing was performed using IMARIS® software version 9.3 (BitPlane). Adobe Photoshop® software version 21.1.1 (Adobe) was used to adjust levels, crop, and resize images.

For salivary gland structural analysis, salivary gland dissection, staining, and confocal imaging were performed as previously described^39^. Salivary glands were stained with DAPI (1:100 in PBS; Thermo Fisher Scientific), Rhodamine-conjugated wheat germ agglutinin (Rh-WGA, 1:67 in PBS, Vector Biosciences), and alpha-tubulin (clone AA4.3) - https://dshb.biology.uiowa.edu/AA4-3 (1:50 in PBS). The samples were incubated with a rabbit anti-TRAP antibody (*P. berghei* repeat region; 1:100; a generous gift from Dr. Photini Sinnis), a mouse anti-CSP antibody (clone 3D11, 1:50, a gift from Dr. Photini Sinnis), and a rabbit anti-cleaved caspase 3 (CC3; 1:50; Cell Signaling, #9661). The secondary antibodies used were goat anti-rabbit IgG conjugated to Alexa Fluor 488 and goat anti-mouse IgG conjugated to Alexa Fluor 647 (Thermo Fisher Scientific, # A-11008 and #A-21235, respectively).

### Mosquito survival, fertility, and fecundity

To assess the survival of transgenic lines, one-day-old adult male and female mosquitoes (*N* = 20) were placed in a cage with cotton pads soaked in 10% syrup solution to feed *ad libitum*. The mosquitoes were kept in the same insectary conditions (temperature, light cycle, humidity) as described above and were monitored daily for mortality. To determine the effect of blood meal on female survival, five-day-old female mosquitoes were placed in cages and allowed to blood feed on an anesthetized mouse for 10 min at day five and day ten after emergence. Mosquitoes were allowed to lay eggs in between blood feedings and were constantly maintained with a sucrose solution. Mortality was monitored daily. Survival percentages were determined as representing the mean survival percentage of three individual biological replicates.

To assess fecundity (number of laid eggs) and fertility (percentage of hatched eggs), five-day-old adult females were blood-fed on anesthetized mice for 20 min. Only fully engorged females were used for these experiments. Two days after blood-feeding, 20 females were placed in individual pints containing a small cup with filter paper soaked in 2 mL of distilled water to stimulate oviposition. After three days (total of five days after blood feeding), the filter paper with eggs was removed, and the number of eggs per mosquito was counted under a stereoscope. After counting, the eggs were placed in paper cups with 50 mL of distilled water to allow hatching for a period of 3 days. Fertility was determined as the number of larvae divided by the total number of eggs. The fecundity and fertility of the transgenic lines were compared to WT mosquitoes, and all the experiments were conducted in three individual biological replicates, except for the Mdg + Sg1 line which was tested in duplicate experiments.

### Quantification of blood uptake

The amount of blood ingested by *An. stephensi* transgenic mosquitoes was determined by measuring the amount of protein-bound heme detected in the mosquito midgut after a blood meal^6^. To determine the amount of heme, transgenic and WT mosquitoes were fed with a 1:1 mixture of plasma and RBCs (Interstate Blood Bank Inc.) by SMFA. After feeding, the midguts of ten fully engorged females were dissected and homogenized individually in 1 mL of distilled water. Unfed mosquitoes were used as the negative control. The spectra reading for protein-bound heme (410 nm) was measured for each individual midgut. The colorimetric assays were measured on a Cytation 5^®^ Cell Imaging Multi-Mode Reader (BioTek Instruments) and recorded with the Gen5 Image Prime software version 3.04. (BioTek Instruments). Colorimetric readings for protein-bound heme were compared among the groups by One-way Anova (*α* = 0.05).

### Transmission-blocking assays

#### P. berghei

Mosquitoes were allowed to feed on an anesthetized Swiss-Webster mouse infected with *P. berghei* ANKA 507m6cl1 expressing GFP (MR4 catalog no. MRA-867,^62^) at 1–2% parasitemia and an exflagellation rate of 1:10 fields. Each transgenic line was simultaneously fed with WT mosquitoes on the same infected mouse, allowing for a direct comparison of parasite infectivity. Mosquito feeding was done for 30 min at 19 °C. Unfed or partially fed mosquitoes were removed, and fully engorged mosquitoes were maintained at 19 °C with 10% corn syrup solution ad libitum. Mosquito midguts were dissected in 1× PBS 12 days post-infection, stained with 0.2% mercurochrome (Sigma-Aldrich) for 30 min, and mature oocysts were counted. For oocyst diameter, mosquito midguts were dissected 12 days post-infection. Oocysts were stained with 0.2% mercurochrome and imaged in an Axio Imager.M2 microscope (Carl Zeiss Microscopy, LLC). Oocyst diameter was measured with the Image-Pro Plus version 4.0 Software.

To determine the number of sporozoites per salivary gland, transgenic and WT mosquitoes simultaneously fed on the same *P. berghei* infected mouse were kept at 19 °C. Salivary glands from 20 mosquitoes were dissected 21 days post-infection, and individually homogenized with a disposable pestle in 50 μL of Schneider′s Insect Medium (Sigma-Aldrich). Sporozoites were counted by loading 10 µL of the homogenate in a disposable hemocytometer (Thermo Fisher Scientific) at 400X magnification on a phase-contrast microscope. The median sporozoite number was compared between each *An. stephensi* transgenic line and the WT. The distribution of parasite numbers in individual mosquitoes between control and experimental groups were compared using nonparametric Mann–Whitney test (GraphPad, Prism). The percent inhibition of median, and prevalence were calculated as follows: 100 × [(number of oocysts in the control − number of oocysts in the experimental)/(number of oocysts in the control)].

#### P. falciparum

Infections were done by standard membrane feeding assay (SMFA) as previously described^63^. Briefly, *P. falciparum* NF54 gametocyte cultures were diluted to 0.2% stage V gametocytemia with O + human RBC at 40% hematocrit in human plasma at 37 °C (Interstate Blood Bank Inc.). The infected blood was added to pre-heated (37 °C) glass membrane feeders and mosquitoes were allowed to feed for 30 min. Unfed and partially fed mosquitoes were removed from the experiment. Mosquitoes were kept with 10% corn syrup solution *ad libitum* until dissection. Mosquito midguts were dissected in 1× PBS 8–9 days post-infection and stained with 0.2% mercurochrome for 30 min. Mercurochrome-stained midguts were counted under the microscope and the infection was recorded.

Inhibition by huPAI-1 was assessed by supplementing the infectious blood meal with increasing concentrations of recombinant huPAI-1 (Sigma Aldrich, # A8111). For the plasmin rescue experiment, infected blood was supplemented with plasmin (Sigma-Aldrich) at a concentration of 100 or 200 µg/mL. The infected blood from control groups was supplemented with an equal volume of PBS. Mosquitoes were maintained and dissected for oocyst counts as described above.

#### P. vivax

Mosquito infections with *P. vivax* were conducted at a NIH Animal Facility 14B North (Bethesda, Maryland, USA), using the non-human primate *Saimiri boliviensis*, according to Sá et al.^64^. Three *S. boliviensis* were infected with the *P. vivax* Chesson strain by cryopreserved or fresh *P. vivax* infected blood inoculated intravenously via the femoral vein^65^. Sixty 3–5-day-old *A. stephensi* females were transferred to individual secure pints and starved for 12 h. After starvation, the mosquitoes were fed for 20 min via directly feeding onto the shaved abdomen of the anesthetized monkey. Mosquito feedings were conducted on alternate days following the monkey parasitemia increase (0.3~1%) in a total of 13 feedings. After feeding, mosquitoes were maintained under standard insectary conditions (described above) and midguts were dissected at day 7 post-feeding for the presence of oocysts in the midgut as described above.

### Sporozoite transmission to mice

Transgenic and WT mosquitoes were simultaneously fed on the same *P. berghei* infected mouse and maintained in the same conditions as described above. Unfed or partially fed mosquitoes were removed from the experiment. A portion of the mosquito midguts was dissected at 10 days post-feeding to determine the infection status by counting the number of oocysts. At day 21 post-feeding, mosquitoes were randomly selected from the cage and were allowed to feed on non-infected mice (challenge). Challenge experiments were done with either one, five, or ten mosquitoes per mouse. Mosquitoes that did not take a blood meal were replaced until the final number of mosquitoes for each group was reached. A total of ten mice were used per experiment, in two biological replicates. After the mosquito challenge, mice were monitored daily for blood-stage infection by Giemsa-stained blood smears during 16 days after the mosquito bite. A blood smear was screened for parasites for five minutes at ×400 magnification.

Statistical significance between control and transgenic lines for the fitness experiments (survival, fertility, and fecundity), such as for the transmission-blocking assays, were defined with α = 0.05 in Prism (Graphpad).

### Statistics

Mosquito survival rates were calculated by Kaplan–Meier survival curves, and multiple comparisons were done by Log-rank test with Bonferroni correction across all lines. Fecundity, fertility and blood ingestion assays were analyzed by Kruskal–Wallis multiple comparisons with Dunn’s post-test. Oocyst or sporozoite inhibition assays were analyzed by Mann–Whitney *U* test For *P. berghei* and Kruskal–Wallis multiple comparisons with Dunn’s posttest for *P. falciparum* and *P. vivax*. Prevalence of infection was analyzed by Fisher’s exact test for *P. berghei* and Chi-squared test for *P. falciparum* and *P. vivax*. Differences were considered significant at *p*  <  0.05. Statistical significance analyses were performed using GraphPad Prism version 9.0. Prepatency data were analyzed by Log-rank (Mantel-Cox) test with *α* = 0.05 and *k* = 0.0166.

### Reporting summary

Further information on research design is available in the Nature Research Reporting Summary linked to this article.

## Supplementary information

Supplementary information

Description of Additional Supplementary Information

Supplementary Data 1

Reporting Summary

## Data Availability

All data needed to evaluate the conclusions are included in the paper and the Supplementary Materials. Source data are provided with this paper.

## Source data

Source Data
