# Supplementary material for: Transgenic Anopheles mosquitoes expressing human PAI-1 impair malaria transmission
Source: Nat Commun. 2022 May 26;13:2949. doi: 10.1038/s41467-022-30606-y (PMC9135733; doi:10.1038/s41467-022-30606-y)
Supplement: Supplementary file 1 — Supplementary information [file 41467_2022_30606_MOESM1_ESM.pdf]

Supplementary Information

TRANSGENIC *ANOPHELES* MOSQUITOES EXPRESSING HUMAN PAI-1 IMPAIR MALARIA TRANSMISSION

Tales V. Pascini[1], Yeong Je Jeong[1], Wei Huang[2], Zarna R. Pala[1], Juliana M. Sá[1], Michael B. Wells[3,4], Christopher Kizito[2], Brendan Sweeney[1], Thiago L. Alves e Silva[1], Deborah J. Andrew[3], Marcelo Jacobs-Lorena[2], and Joel Vega-Rodriguez[1,*]

[1] Laboratory of Malaria and Vector Research, National Institute of Allergy and Infectious Diseases, National Institutes of Health, 12735 Twinbrook Parkway, Rm 2E20A, Rockville, MD 20852, USA.
[2] Department of Molecular Microbiology and Immunology, Malaria Research Institute, Johns Hopkins Bloomberg School of Public Health, Baltimore, MD 21205, USA.
[3] Department of Cell Biology, Johns Hopkins University School of Medicine, 725 N. Wolfe Street, G10 Hunterian, Baltimore, MD 21205, USA.
[4] Current address: Department of Biomedical Sciences, Idaho College of Osteopathic Medicine, Meridian, ID 83642.

*Corresponding author: joel.vega-rodriguez@nih.gov

Supplementary Figures and Figure Legends

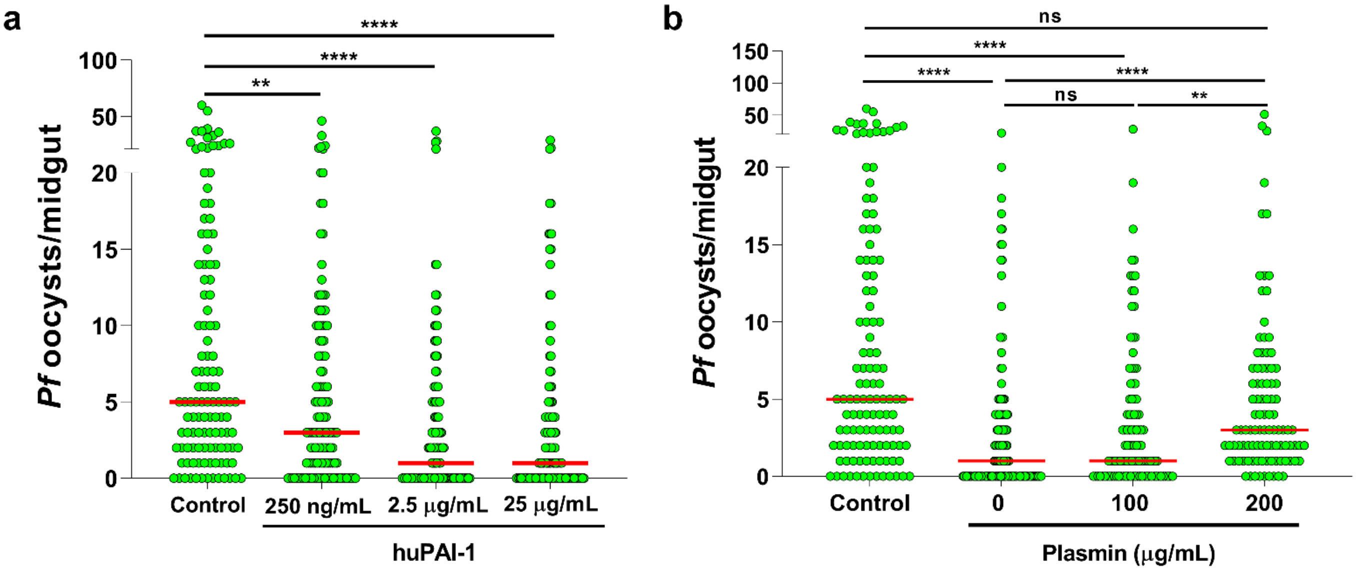

	Control	250 ng/mL	2.5 μg/mL	25 μg/mL
N	118	128	126	131
Median	5	3	1	1
I median (%)	–	40	80	80
Prevalence (%)	88	75	54	56
I prevalence (%)	–	15	38	36

	Control	0	100	200
N	118	137	108	98
Median	5	1	1	3
I median (%)	–	80	80	–
Prevalence (%)	88	51	67	90
I prevalence (%)	–	41	23	–

Supplementary Fig. 1. huPAI-1 inhibits *P. falciparum* infection of *An. stephensi*. a, b Oocysts numbers were determined in WT *An. stephensi* mosquitoes after feeding on *P. falciparum* infectious blood supplemented with increasing concentrations of huPAI-1 (**a**) or with 250 ng/mL of huPAI-1 plus plasmin (**b**). Horizontal red lines represent the median oocyst number of data pooled from at least three independent experiments shown in Supplementary Data 1, Datasets 1 and 2. Statistical analysis was done by Kruskal-Wallis multiple comparisons with Dunn’s posttest. **a** **P=0.0036, ****P<0.0001. **b** **P=0.0022, ****P<0.0001. I- % inhibition, N- number of analyzed mosquitoes. The percentage inhibition of median, and prevalence was calculated as follows: 100 x [(number of oocysts in the control – number of oocysts in the experimental)/ (number of oocysts in the control)].

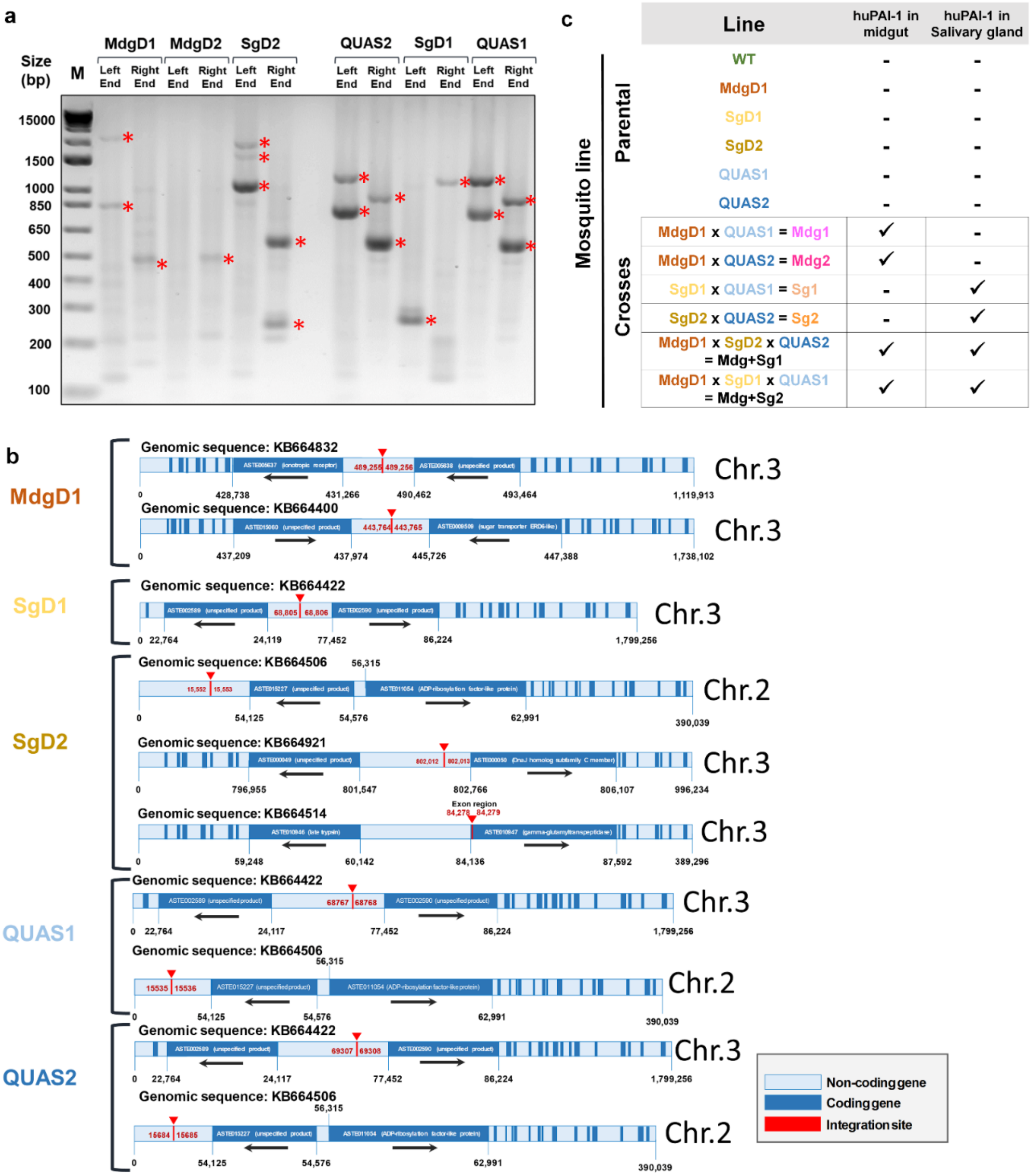

Supplementary Fig. 2. Mapping of plasmid genome integration sites. a Representative agarose gels showing the splinkerette PCR products to map the insertion 5′ or 3′ sequences. Bands were excised and sequenced. M- marker. The MdgD2 line was not used for the experiments described in this report. Data representative of two independent experiments. **b** Localization of plasmid integration sites in the An. stephensi genome. Black arrows indicate the open reading frame orientation of the genes flanking the integration site. Chr- chromosome. **c** List of the parental lines and the crosses to obtain the transgenic lines expressing huPAI-1 in the midgut and/or the salivary glands. Check marks points to expected expression of huPAI-1 in each tissue and line.

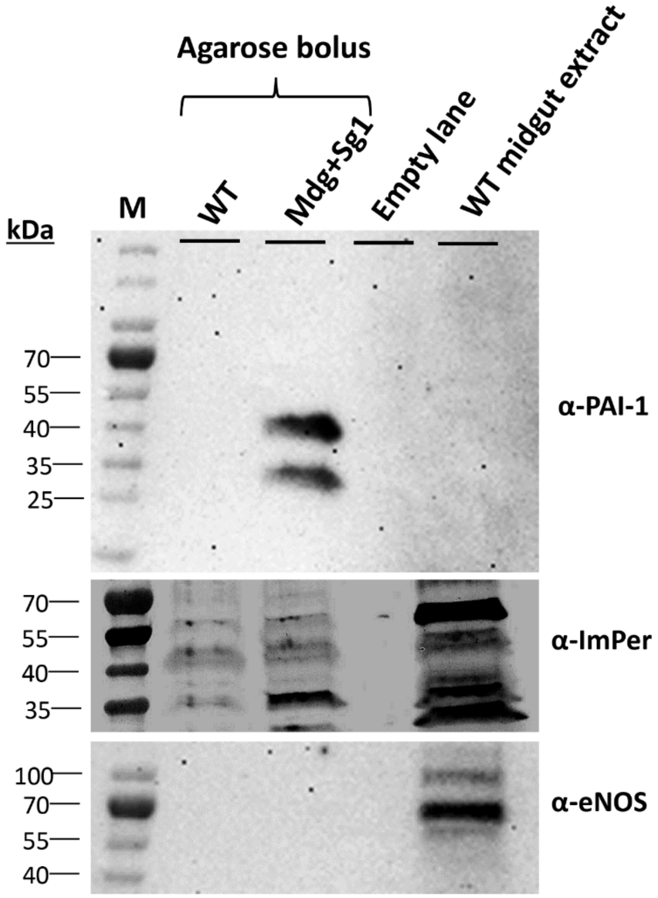

Supplementary Fig. 3. Secretion of huPAI-1 into the midgut lumen of transgenic mosquitoes. Secretion of huPAI-1 in the midgut lumen was assessed by the low-melting agarose assay. Liquid low-melting agarose was fed to mosquitoes and after solidification, agarose boluses were dissected, and the secreted proteins trapped in the bolus were analyzed by Western blotting with an anti-huPAI-1 antibody. An anti-IMPer antibody was used as a positive control for a midgut secreted protein and an anti-eNOS was used as a negative control for a midgut non-secreted protein. The MDG+Sg1 line was used as representative for the transgenic lines. M- marker, WT-wild type. Data representative of two independent experiments.

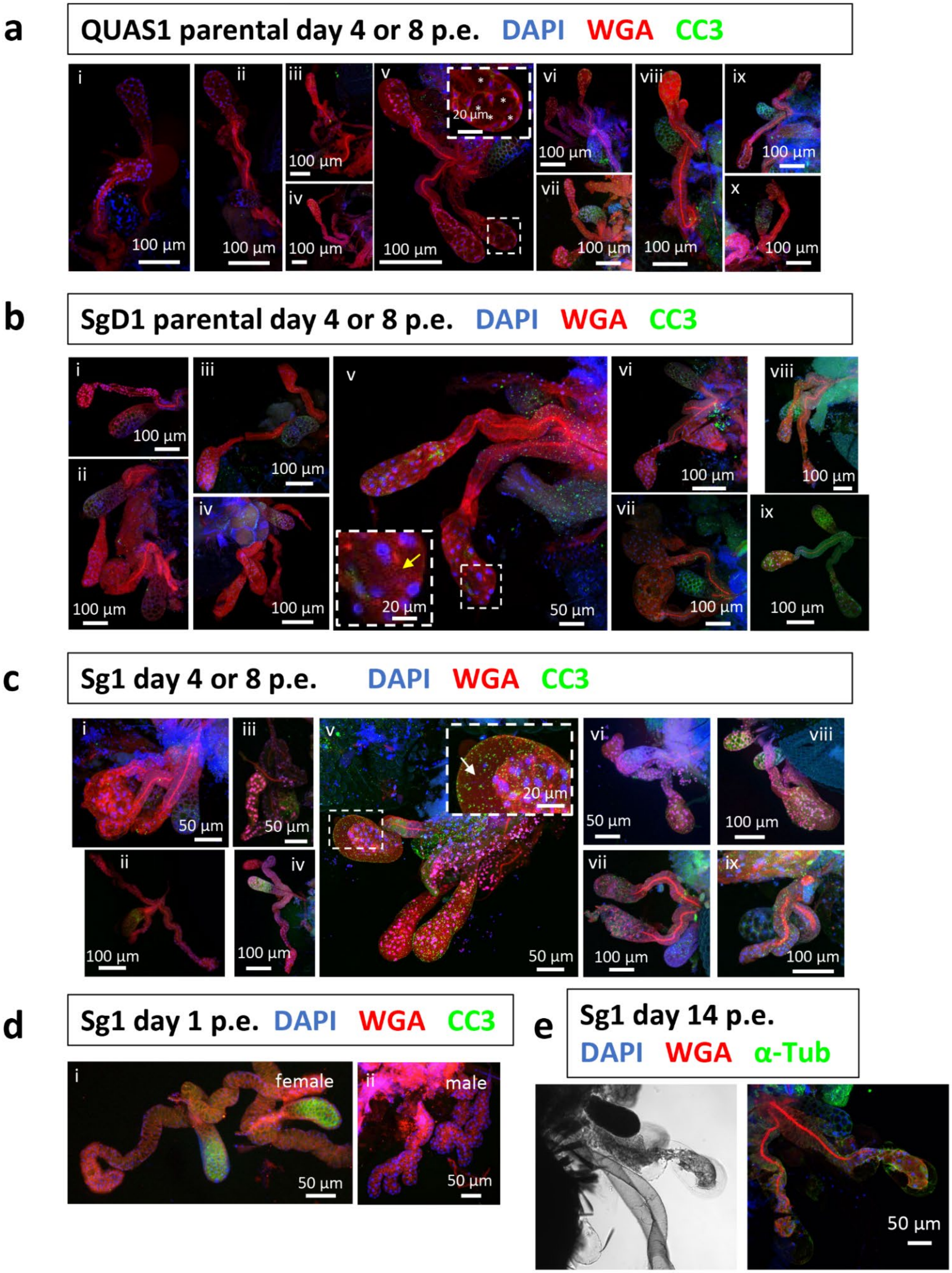

a
QUAS1 parental day 4 or 8 p.e. DAPI WGA CC3
i
ii
iii
iv
v
vi
vii
viii
ix
x
20 µm
100 µm
b
SgD1 parental day 4 or 8 p.e. DAPI WGA CC3
i
ii
iii
iv
v
vi
vii
viii
ix
20 µm
50 µm
100 µm
c
Sg1 day 4 or 8 p.e. DAPI WGA CC3
i
ii
iii
iv
v
vi
vii
viii
ix
20 µm
50 µm
100 µm
d
Sg1 day 1 p.e. DAPI WGA CC3
female
male
50 µm
e
Sg1 day 14 p.e.
DAPI WGA α-Tub
50 µm

Supplementary Fig. 4. huPAI-1 expression leads to altered salivary gland structure. Salivary glands (SGs) were dissected four or eight days (**a-c**), one day (**d**), or fourteen days **(e)** post-emergence and stained with anti-cleaved caspase 3 antiserum (CC3, a cell death marker; green in panels **a-d**), anti-α-tubulin (α-Tub, green in panel **e**), DAPI (DNA, blue), and WGA (chitin/O-GlcNAcylation; red). Shown are representative 3D projection (MIP) confocal images of parental female QUAS-PAI-1 effector (**a**), parental female SgD1 driver (**b**), and male or female huPAI Sg transgenic (**c**-**d**) SGs. SGs are organized left to right by increasing CC3 signals in the lateral lobes. SGs from huPAI Sg transgenic and QUAS-PAI-1 males have very little CC3 staining confined to a perinuclear localization (data not shown). CC3 staining of female huPAI Sg transgenic medial lobe is high even at day 1 post-emergence (Di), but low and diffused in the lateral lobes, which possess a WT SG architecture at this early time point compared to day 4 or 8 post-emergence (**a**-**c**). No phenotypic differences were observed between day 4 and day 8 adult SGs. The parental SgD1 driver and huPAI Sg transgenics (**b**-**c**) consistently show lumen and secretory cavity loss, cell misorganization, and variable (but higher) levels and intensities of CC3 punctae than QUAS-PAI-1 females **(a)**. For example, compare Bv and Cv to Av (figures and inserts): asterisks mark secretory cavities (Av); yellow arrows mark lumen loss (Bv) or cell misorganization (Bv, Cv); and white arrow mark cells pulling away from basement membrane (Cv). Substantial damage to the distal lobes can be observed at day 14 post emergence **(e)**. Refer to **Fig. 2** for phenotypic explanations. Data in panels a-e is representative of at least two independent experiments.

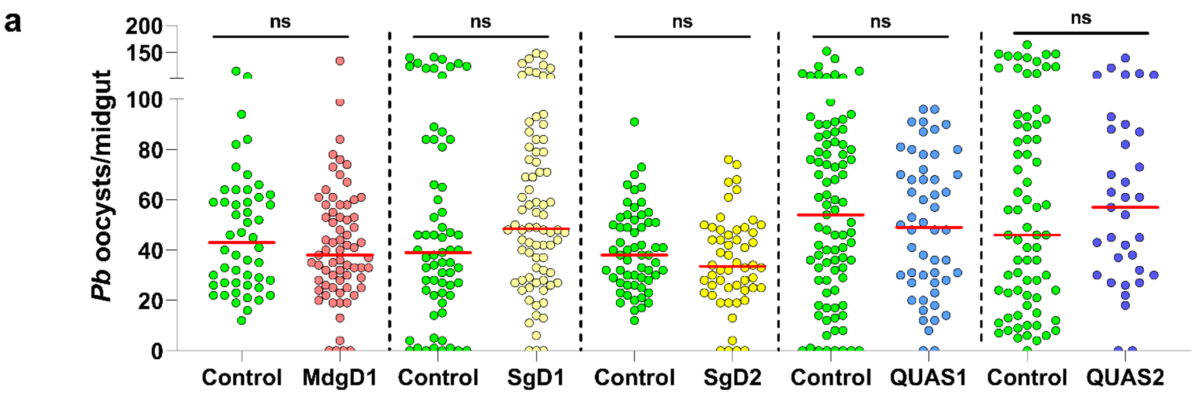

a

	Control	MdgD1	Control	SgD1	Control	SgD2	Control	QUAS1	Control	QUAS2
N	50	69	65	78	58	54	89	52	73	35
Median	43	38	39	48	38	33	54	49	46	57
Prevalence (%)	100	92	92	96	100	92	92	96	98	94

b

	Control	MdgD1	Control	SgD1	Control	SgD2	Control	QUAS1	Control	QUAS2
N	29	34	35	40	40	34	40	32	34	25
Median	9625	9687	10125	9875	5875	5437	7437	5406	5406	5437
Prevalence (%)	96	94	97	97	97	100	97	100	100	100

Supplementary Fig. 5. Midgut and salivary gland infection are not affected in the transgenic parental lines. WT and transgenic parental mosquitoes were simultaneously fed on the same *P. berghei* infected mouse (WT and one transgenic per infected mouse). **a** Midgut infections were determined by oocyst counts at 10 days post-infection. **b** Salivary gland infection was determined by the total number of sporozoites present in the salivary gland of individual mosquitoes at 21 days post-infection. Horizontal red lines represent the medians. Oocyst data pooled from three independent experiments and salivary gland data pooled from two independent experiments (Supplementary Data 1, Datasets 3 and 7). Statistical analysis was done by two-tailed Mann-Whitney U test. ns: not significant, N: number of analyzed mosquitoes.

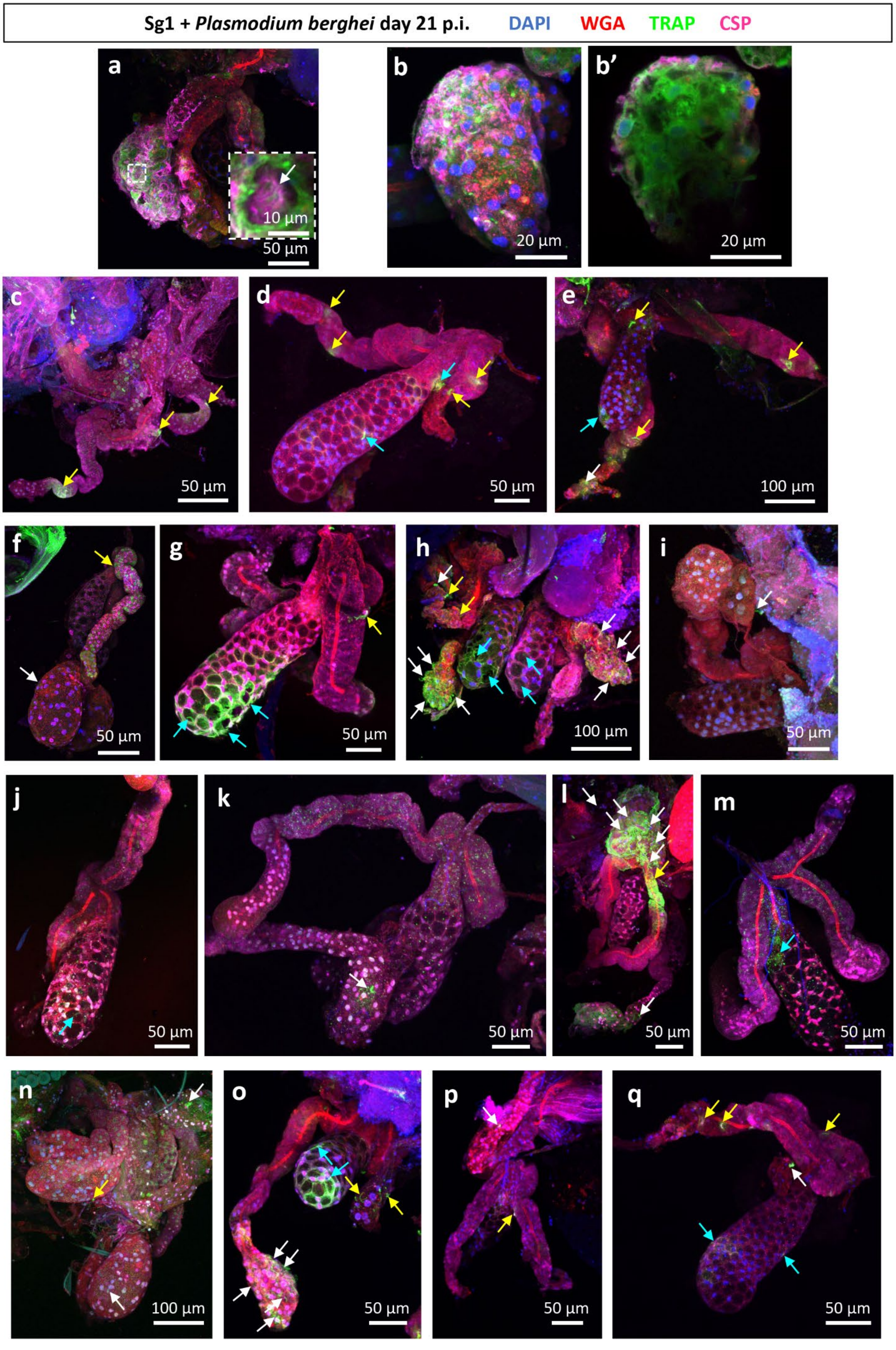

Sg1 + *Plasmodium berghei* day 21 p.i.
DAPI
WGA
TRAP
CSP
a
10 µm
50 µm
b
20 µm
b'
20 µm
c
50 µm
d
50 µm
e
100 µm
f
50 µm
g
50 µm
h
100 µm
i
50 µm
j
50 µm
k
50 µm
l
50 µm
m
50 µm
n
100 µm
o
50 µm
p
50 µm
q
50 µm

Supplementary Fig. 6. Poor sporozoite invasion and few accumulations are observed in huPAI-1 Sg transgenics. Salivary glands from transgenic mosquitoes expressing huPAI-1 in the salivary glands were dissected 21 days post infection, processed, and stained with anti-TRAP (parasite; green) and anti-CSP (parasite; magenta) antisera, DAPI (DNA, blue), and WGA (chitin/O-GlcNAcylation; red). Invasion events are marked by lobe type using colored arrows (DL-white, PL-yellow, M-cyan). Results suggest that SGs expressing huPAI-1 are consistently poorly invaded by *Plasmodium berghei* sporozoites. High accumulations of shed parasite proteins within some DL lobes, in the absence of large numbers of invaded parasites [**b, b'**, TRAP (green)], may indicate that some invaded sporozoites die.

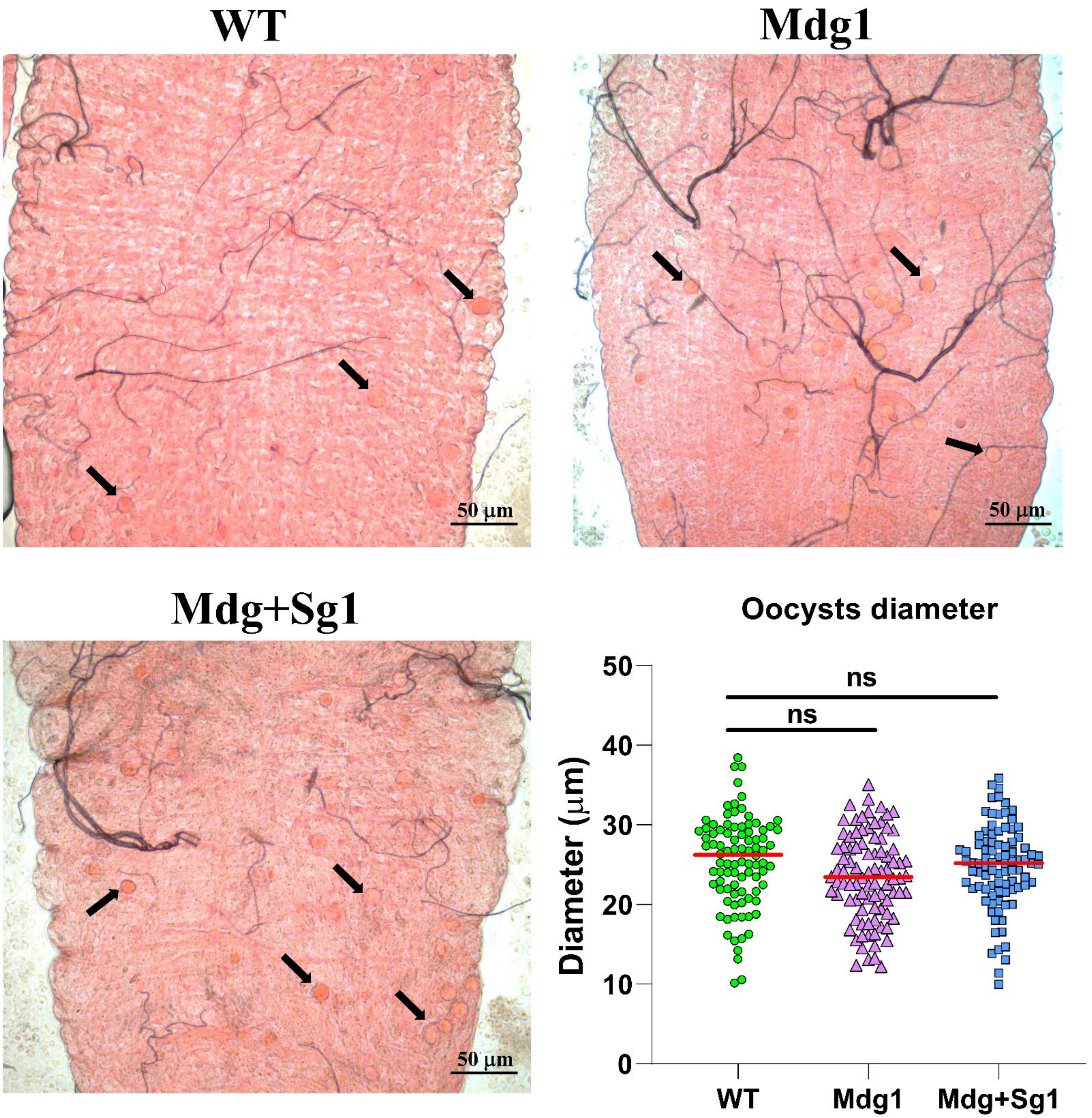

Supplementary Fig. 7. *P. berghei* oocyst diameter in WT and transgenic lines. Oocyst diameter was measured in *P. berghei* infected midguts from WT, Mdg1 and Mdg+Sg1 mosquitoes dissected 12 days-post infection. Oocysts were stained with 0.2% mercurochrome and imaged in an Axio Imager.M2 microscope (Carl Zeiss Microscopy, LLC). Oocyst diameter was measured with the Image-Pro Plus version 4.0 software. Black arrows point at oocysts. Statistical analysis was done by Kruskal-Wallis multiple comparisons with Dunn's posttest. Data from two independent experiments. ns: not significant.

Supplementary Data 1. Analysis of individual mosquito infections with *P. berghei*, *P. falciparum* and *P. vivax*.

Provided as a separate excel file.

Supplementary Table 1. Quantification of architecture and sporozoite organization in SGs of WT and Sg1 transgenic mosquitoes. Salivary glands were dissected 21 days post infection with *P. berghei* parasites and were stained with anti-TRAP (parasite) and anti-CSP (parasite) antisera, DAPI (DNA), and WGA (chitin/O-GlcNAcylation). The data includes the phenotypes from salivary glands of WT and Sg1 transgenic mosquitoes shown in Fig. 6B-C and Supplementary Fig. 6.

Strain/lobe	# with good architecture	# infected	# with spz bundles	# with spz in lumen
WT DL (n=8)	7 (88%)	7 (88%)	7 (88%)	7 (88%)
WT PL (n=9)	8 (89%)	5 (63%)	0	0
WT M (n=9)	3 (33%)	2 (22%)	0	1 (11%)
Sg1 DL (n=33)	6 (18%)	18 (55%)	8 (24%)	3 (9%)
Sg1 PL (n=46)	14 (30%)	19 (41%)	3 (6.5%)	0
Sg1 M (n=21)	15 (71%)	8 (38%)	2 (9.5%)	3 (14%)

Supplementary Table 2. List of primers used in this study.

Primers	Sequence
**PAI-FW**	5’ - GCGGCCGCGGCTCGAG ATGCAGATGTCTCCAGCCCT – 3’
**PAI-RV**	5’ – AGATCGACGTCTCGAGTCAGGGTTCCATCACTTGGC – 3’
**MgP-FW**	5’ – ATCAATGTATCTCGAGTACCGGCAATACTGGTTGTTGAGG – 3’
**MgP-RV**	5’ – GTTGGCCGGCCTCGAGGATGAGAATGTTAGATGCCGCGAGTTG – 3’
**YFP-FW**	5’ – GGGCCCGGGATCCACCGGTCGCCACCATGGTGAGCAAGGGCGAGGA – 3’
**YFP-RV**	5’ – GCGGCCGCTACTTGTACAGCTCGTCCA – 3’
**SgP - FW**	5’ – ATCAATGTATCTCGAGGGACTTCGCGTCGGTAGTAG – 3’
**SgP - RV**	5’ – GTTGGCCGGCCTCGAGCGTTTATTCACCTGTGAGCTATGG – 3’
**splink-Top**	**5`-** GATCCCACTAGTGTCGACACCAGTCTCTAATTTTTTTTTTCAAAAAAA **- 3`**
**splink-Bottom**	**5`-** CGAAGAGTAACCGTTGCTAGGAGAGACCGTGGCTGAATGAGACTGGTGTCGACACTAGTGG **- 3`**
**splink#1**	**5` -** CGAAGAGTAACCGTTGCTAGGAGAGACG **- 3`**
**splink #2**	**5`-** GTGGCTGAATGAGACTGGTGTCGAC **- 3`**
**piggybac LE#1**	**5`-** CAGTGACACTTACCGCATTGACAAGC **- 3`**
**piggybac LE#2**	**5` -** GCGACTGAGATGTCCTAAATGCAC **- 3`**
**piggybac RE#1**	**5` -** CGATATACAGACCGATAAAACACATGCGTC **- 3`**
**piggybac RE#2**	**5` -** ACGCATGATTATCTTTAACGTAC **- 3`**
**huPAI-1 - FW**	**5`-** TCCAGCGGGATCTGAAGCTG **- 3`**
**huPAI-1 - RV**	**5` -** TGTCCCAAGCAAGTGGCTGA **- 3`**
**S7 - FW**	**5’ -** CTAACGACACGAAGACCACAAGA **- 3’**
**S7 - RV**	**5’ -** CAACCTGCAACGAAGCAAAA **- 3’**
