# Supplementary material for: Transgenic Anopheles mosquitoes expressing human PAI-1 impair malaria transmission
Source: Nat Commun. 2022 May 26;13:2949. doi: 10.1038/s41467-022-30606-y (PMC9135733; doi:10.1038/s41467-022-30606-y)
Supplement: Supplementary file 2 — Description of Additional Supplementary Information [file 41467_2022_30606_MOESM2_ESM.docx]

Description of additional supplementary data

Item: Supplementary Data 1

Description: Analysis of individual mosquito infections with *P. berghei*, *P. falciparum* and *P. vivax*.
