# Supplementary material for: Transgenic Anopheles mosquitoes expressing human PAI-1 impair malaria transmission
Source: Nat Commun. 2022 May 26;13:2949. doi: 10.1038/s41467-022-30606-y (PMC9135733; doi:10.1038/s41467-022-30606-y)
Supplement: Supplementary file 4 — Reporting Summary [file 41467_2022_30606_MOESM4_ESM.pdf]

nature portfolio

Corresponding author(s): Joel Vega-Rodríguez

Last updated by author(s): Jan 20, 2022

Reporting Summary

Nature Portfolio wishes to improve the reproducibility of the work that we publish. This form provides structure for consistency and transparency in reporting. For further information on Nature Portfolio policies, see our Editorial Policies and the Editorial Policy Checklist.

Statistics

For all statistical analyses, confirm that the following items are present in the figure legend, table legend, main text, or Methods section.

n/a	Confirmed	
☐	☒	The exact sample size (n) for each experimental group/condition, given as a discrete number and unit of measurement
☐	☒	A statement on whether measurements were taken from distinct samples or whether the same sample was measured repeatedly
☐	☒	The statistical test(s) used AND whether they are one- or two-sided<br>*Only common tests should be described solely by name; describe more complex techniques in the Methods section.*
☐	☒	A description of all covariates tested
☐	☒	A description of any assumptions or corrections, such as tests of normality and adjustment for multiple comparisons
☐	☒	A full description of the statistical parameters including central tendency (e.g. means) or other basic estimates (e.g. regression coefficient) AND variation (e.g. standard deviation) or associated estimates of uncertainty (e.g. confidence intervals)
☐	☒	For null hypothesis testing, the test statistic (e.g. F, t, r) with confidence intervals, effect sizes, degrees of freedom and P value noted<br>*Give P values as exact values whenever suitable.*
☒	☐	For Bayesian analysis, information on the choice of priors and Markov chain Monte Carlo settings
☒	☐	For hierarchical and complex designs, identification of the appropriate level for tests and full reporting of outcomes
☒	☐	Estimates of effect sizes (e.g. Cohen's d, Pearson's r), indicating how they were calculated

Our web collection on statistics for biologists contains articles on many of the points above.

Software and code

Policy information about availability of computer code

Data collection	Data for microscopy imaging were collected on a Leica SP8 DMI 6000 (Leica Microsystems).<br>Spectophotometry data were collected on a Cytation 5 ® Cell Imaging Multi-Mode Reader (BioTek Instruments).<br>Flow cytometry data was collected on a Amnis ImageStreamX Mark II flow cytometer (Luminex).<br>Western blot data was collected on an Azure Imager c600.
Data analysis	Microscopy image processing was performed using Imaris 9.3 (Bitplane).<br>Data for spectophotometry assays were analyzed with the Gen5 Image Prime software version 3.04. (BioTek Instruments)<br>Western blot data was analyzed using the Gen5 Image Prime software version 3.04. (BioTek Instruments)<br>Statistical analyses were done using Graphpad Prism version 9.0<br>Adobe Photoshop® 2021 software

For manuscripts utilizing custom algorithms or software that are central to the research but not yet described in published literature, software must be made available to editors and reviewers. We strongly encourage code deposition in a community repository (e.g. GitHub). See the Nature Portfolio guidelines for submitting code & software for further information.

Data

Policy information about availability of data

All manuscripts must include a data availability statement. This statement should provide the following information, where applicable:

- Accession codes, unique identifiers, or web links for publicly available datasets
- A description of any restrictions on data availability
- For clinical datasets or third party data, please ensure that the statement adheres to our policy

All data needed to evaluate the conclusions are included in the paper and the Supplementary Materials. Source data are provided with this paper.

Field-specific reporting

Please select the one below that is the best fit for your research. If you are not sure, read the appropriate sections before making your selection.

[x] Life sciences [ ] Behavioural & social sciences [ ] Ecological, evolutionary & environmental sciences

For a reference copy of the document with all sections, see nature.com/documents/nr-reporting-summary-flat.pdf

Life sciences study design

All studies must disclose on these points even when the disclosure is negative.

Sample size	No statistical methods were used to determine sample size. Sample size was determined to be adequate based on experimental consistency and effect, and previous publications in a similar manner as in Alves e Silva et al. Sci Adv. 2021 Feb 5;7(6):eabe3362. Generally three independent biological replicates were done for each experiment. For transmission blocking assays, at least 20 female mosquitoes were included per experiment in at least three biological replicates. Twenty mosquitoes were used for each experiment of mosquito survival, fertility, fecundity and blood ingestion in at least two biological replicates. For microscopy, at least 5-10 mosquitoes were imaged for each experiment performed at least twice independently.
Data exclusions	No data were excluded from analysis
Replication	All replicates were successful and included in data analysis as indicated in the manuscript and as shown in the Supplementary Table 1. PCRs and Western blots were repeated at least twice independently with at least 10 mosquitoes processed for each individual sample. Imaging experiments were performed at least twice, independently, for each line analyzed. Experiments for mosquito survival, fertility, fecundity and blood ingestion were performed in two or more biological replicates.
Randomization	All mice or mosquitoes used in this study were pooled from different cages and were randomly distributed into control and experimental groups. For the rest of the experiments randomization does not apply.
Blinding	The investigators were not blinded to the allocation during the experiments or to the outcome assessment. The same researcher that set up the experiments also collects the data. Unbiased experimental procedure and data analysis were carried out when possible.

Reporting for specific materials, systems and methods

We require information from authors about some types of materials, experimental systems and methods used in many studies. Here, indicate whether each material, system or method listed is relevant to your study. If you are not sure if a list item applies to your research, read the appropriate section before selecting a response.

Materials & experimental systems

n/a	Involved in the study	
[ ]	[x]	Antibodies
[x]	[ ]	Eukaryotic cell lines
[x]	[ ]	Palaeontology and archaeology
[ ]	[x]	Animals and other organisms
[ ]	[x]	Human research participants
[x]	[ ]	Clinical data
[x]	[ ]	Dual use research of concern

Methods

n/a	Involved in the study	
[x]	[ ]	ChIP-seq
[x]	[ ]	Flow cytometry
[x]	[ ]	MRI-based neuroimaging

Antibodies

Antibodies used	anti-human PAI-1 antibody (validated by BD Biosciences, clone 41/PAI-1, #612024)<br>anti-mouse HRP-linked antibody (validated byCell Signaling, #7076)<br>anti-AAPP antibody (validated by Hayashi et al., 2012)

	anti-heme peroxidase IMPer (provided and validated by Dr. Carolina Barillas-Mury, NIAID, NIH)<br>rabbit anti-TRAP antibody (provided and validated by Dr. Photini Sinnis)<br>mouse anti-CSP antibody (clone 3D11, validated by Dr. Photini Sinnis)<br>rabbit anti-cleaved caspase 3 (CC3; validated byCell Signaling 9661S)<br>rabbit anti-mouse HRP-linked antibody (Cell Signaling, # 7074)<br>goat anti-mouse IgG conjugated to Alexa Fluor 594 (validated by ThermoFisher Scientific, # A-11005)<br>goat anti-rabbit IgG conjugated to Alexa Fluor 488 (validated by ThermoFisher Scientific, # A-11008)<br>goat anti-mouse IgG conjugated to Alexa Fluor 647 (validated by ThermoFisher Scientific, # A-21235)<br>Alexa Fluor™ 647 Phalloidin (validated by ThermoFisher Scientific, # A-22287)
Validation	anti-human PAI-1 antibody (validated by BD Biosciences in Human (QC Testing), Mouse, Rat, Dog, Chicken, clone 41/PAI-1, #612024)<br>anti-mouse HRP-linked antibody (validated byCell Signaling, #7076)<br>anti-AAPP antibody (validated by Hayashi et al., 2012)<br>anti-heme peroxidase IMPer (provided and validated in Anopheles mosquitoes by Dr. Carolina Barillas-Mury, NIAID, NIH)<br>rabbit anti-TRAP antibody (provided and validated by Dr. Photini Sinnis)<br>mouse anti-CSP antibody (clone 3D11, validated by Dr. Photini Sinnis)<br>rabbit anti-cleaved caspase 3 (CC3; validated byCell Signaling 9661S)<br>rabbit anti-mouse HRP-linked antibody (Cell Signaling, # 7074)<br>goat anti-mouse IgG conjugated to Alexa Fluor 594 (validated by ThermoFisher Scientific, # A-11005)<br>goat anti-rabbit IgG conjugated to Alexa Fluor 488 (validated by ThermoFisher Scientific, # A-11008)<br>goat anti-mouse IgG conjugated to Alexa Fluor 647 (validated by ThermoFisher Scientific, # A-21235)<br>Alexa Fluor™ 647 Phalloidin (validated by ThermoFisher Scientific, # A-22287)

Animals and other organisms

Policy information about studies involving animals; ARRIVE guidelines recommended for reporting animal research

Laboratory animals	Anopheles stephensi, Liston strain (Feldmann et al., 1989), Female, 3-21 days old<br>Swiss webster mice, Female, 4-6 weeks old, NIAID animal study proposals LMVR-22. Mice were kept at 72 ± 3 °F, 30-70% humidity and a light/dark cycle of 14 hours of light and 10<br>hours of dark.<br>Saimiri boliviensis, NIAID animal study proposals LMVR-9: female 1 born on 5/25/2008, 11 years old; female 2 born on 5/09/2009, 10 years old.
Wild animals	The study did not involve wild animals
Field-collected samples	The study did not involve wild animals
Ethics oversight	Studies with animals were approved by the Institutional Animal Care and Use Committee (IACUC), Care and Use Committees (NIAID ACUC)<br>Swiss webster mice, Female, 4-6 weeks old, NIAID animal study proposals LMVR-22<br>Saimiri boliviensis, NIAID animal study proposals LMVR-9

Note that full information on the approval of the study protocol must also be provided in the manuscript.

Human research participants

Policy information about studies involving human research participants

Population characteristics	Blood obtained from donors was used for P. falciparum culturing and mosquito feeding. Donors must meet the eligibility criteria for volunteer blood donation, defined in the Code of Federal Regulations 21 CFR 640 and AABB Standards as modified in the 2007 FDA/CBER Guidance Document: Eligibility Criteria for Donors of Human Cells, Tissues, and Cellular and Tissue-Based Products with the exception of foreign travel history and other conditions.
Recruitment	Donors are volunteers and were evaluated and selected for study participation by DTM apheresis nurses at a "pre-assessment" session during which a health history screen, a mini-physical consisting of general<br>appearance and vital signs, bilateral antecubital venous access assessment, and blood samples for CBC (complete blood count) and infectious disease marker (IDM) testing are obtained.
Ethics oversight	Human blood for P. falciparum culturing was collected from healthy volunteers at the NIH Clinical Center Blood Bank. All individuals gave written informed consent and enrolled in a protocol approved by the National Institutes of Health Clinical Center Institutional Review Board (NIH protocol 99-CC-0168 "Collection and Distribution of Blood Components from Healthy Donors for In Vitro Use").

Note that full information on the approval of the study protocol must also be provided in the manuscript.
